# The Nervous Systems of Basally Branching Nemertea (Palaeonemertea)

**DOI:** 10.1371/journal.pone.0066137

**Published:** 2013-06-13

**Authors:** Patrick Beckers, Rudi Loesel, Thomas Bartolomaeus

**Affiliations:** 1 Institute for Evolutionary Biology and Ecology, Rheinische Friedrich-Wilhelms-Universität Bonn, Bonn, Germany; 2 Unit of Developmental Biology and Morphology of Animals, Institute for Biology II, RWTH Aachen University, Aachen, Germany; University of Maryland, United States of America

## Abstract

In recent years, a lot of studies have been published dealing with the anatomy of the nervous system in different spiralian species. The only nemertean species investigated in this context probably shows derived characters and thus the conditions found there are not useful in inferring the relationship between nemerteans and other spiralian taxa. Ingroup relationships within Nemertea are still unclear, but there is some agreement that the palaeonemerteans form a basal, paraphyletic grade. Thus, palaeonemertean species are likely the most informative when comparing with other invertebrate groups. We therefore analyzed the nervous system of several palaeonemertean species by combining histology and immunostaining. 3D reconstructions based on the aligned slices were performed to get an overall impression of the central nervous system, and immunohistochemistry was chosen to reveal fine structures and to be able to compare the data with recently published results. The insights presented here permit a first attempt to reconstruct the primary organization of the nemertean nervous system. This comparative analysis allows substantiating homology hypotheses for nerves of the peripheral nervous system. This study also provides evidence that the nemertean brain primarily consists of two lobes connected by a strong ventral commissure and one to several dorsal commissures. During nemertean evolution, the brain underwent continuous compartmentalization into a pair of dorsal and ventral lobes interconnected by commissures and lateral tracts. Given that this conclusion can be corroborated by cladistic analyses, nemerteans should share a common ancestor with spiralians that primarily have a simple brain consisting of paired medullary, frontally commissurized and reinforced cords. Such an organization resembles the situation found in presumably basally branching annelids or mollusks.

## Introduction

Nemerteans are a spiralian taxon, of which most described species are marine benthic hunters. The majority of the animals are nocturnal predators able to follow their prey [1], [2], [3] and catch it with a unique structure, the eversible proboscis. Due to their lifestyle, nemerteans possess well-developed nervous systems and sensory structures, enabling them to interact with their environments. Since nearly all nemerteans are predators, their central nervous system has been considered quite uniform, composed of a four-lobed brain and two lateral medullary cords which originate from the ventral lobes of the brain and extend the whole length of the animal until they converge at the posterior tip of the animal [4], [5]. The paired dorsal and ventral lobes of the brain are interconnected by a commissural tract above or below the rhynchocoel. The central nervous system is composed of a central neuropil that is homogeneously surrounded by neuronal cell somata. The neuronal cell somata may be separated by an extracellular matrix, the inner neurilemma, from the neuropil and/or the entire brain may be enclosed by an outer neurilemma [4], [6], [7]. A set of minor nerves, such as the cephalic, esophageal and proboscidial nerves originate from different parts of the brain, while additional ventral and dorsal nerves are only found in certain groups. These minor nerves are not covered by neuronal cell somata (perikarya) and belong to the peripheral nervous system.

Nemerteans possess a number of sensory organs, which are mostly located on the tip of the head. These organs are called the frontal and cerebral organs and are supposed to be involved in chemoreception [8], [9], [10], [11], [12]. Photoreception is perceived through pigment cup eyes, of which up to hundred may be present in one animal [5]. Adult palaeonemerteans generally lack eyes although larval eyes have been reported for two species (e. g. *Procephalothrix simulus* [12] and *Carinoma tremaphoros* [13]).

Formerly, nemerteans were classified in three higher taxa: Palaeo-, Hetero- and Hoplonemertea [4], [15]. Palaeonemerteans were regarded as the basal most branching taxon of nemerteans [15], [16] [17]. Traditionally, four different taxa belonged to this group, the Tubulanidae, Cephalothricidae, Carinomidae and Hubrechtidae [4], [5], [14]. New evidence however, suggests that *Hubrechtidae* are the sister group of Heteronemertea, because they possess a pilidium larvae and a cerebral organ which is typical for heteronemerteans [16].

Recent molecular studies provide some evidence that the remaining palaeonemerteans could be a monophyletic group [17]. Morphological characters supporting this result, however, are missing. *Cephalothricidae* have been suggested as the basal most branching nemertean group due to the absence of cerebral organs and the position of the nervous system inside the musculature [18]. Thus, palaeonemerteans form a basal grade rather than a basally branching clade of nemerteans, a conclusion that has also been drawn by earlier morphologists [4] and molecular studies [15], [16], [17].

The palaeonemertean nervous system shows the highest variation among nemertean taxa [4], [5] and potentially provides a rich source of characters. In recent years, neuroanatomy, by means of immunohistochemistry, has yielded promising data that contributes to unraveling the phylogeny of certain metazoan groups, e.g [19], [20], [21], [22], [23]. The only representative of nemerteans investigated with this method so far, *Lineus viridis* [24], probably shows derived characters and data concerning basal branching nemerteans is still lacking. Studies on the phylogeny of Nemertea and the relationship they share with other lophotrochozoans need to focus particularly on basally branching nemertean taxa. Therefore, we started a comparative analysis of the nemertean nervous system [25]. In this study, the first of a series of papers, we are presenting detail information on the neuroanatomy of different species of the palaeonemerteans. Classical Azan staining was chosen to provide an entire view of the elements present in the nervous system and for the inference of the position of the central nervous system in relation to the body wall layers. Additionally, aligned slices that were used to generate 3D reconstructions of the whole central nervous system are made freely available in MorphDBase, a morphological database [26] (www.morphdbase.de), in terms of data transparency. The respective hyperlink is given at the beginning of each description. Immunochemistry was chosen to reveal the fine structure of nervous system. Antibody subsets representing standard markers that are used by many researchers and allow comparability of the morphological elements are also chosen here. Given that palaeonemerteans are likely not monophyletic and comprise an assemblage of basally branching nemerteans, some hypotheses on the primary organization of the nemertean nervous system can be derived and evaluated with respect to certain outgroups.

## Results

### General remarks

**Figure 1:**
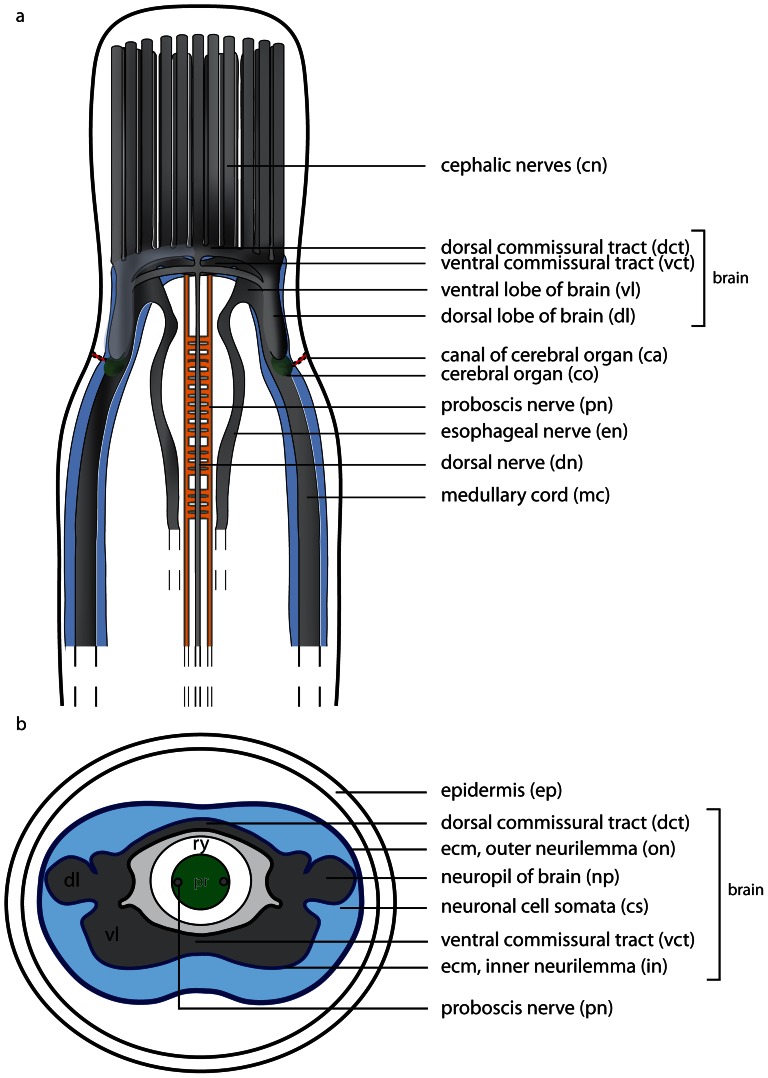
Schematic drawings of the nemertean central nervous system. **a**: Dorsal view of the central nervous system, the neuronal cell somata are shown in *bright blue*. **b**: Transverse section of the brain. The brain is composed of a central neuropil (*gray*), which is surrounded by neuronal cell somata (*bright blue*). The neuronal cell somata may be separated from the neuropil by an inner neurilemma. The whole brain may be enclosed by an outer neurilemma. The two halves of the brain are interconnected by a dorsal (*dct*) and a ventral commissural tract (*vct*) which form a ring around the rhynchocoel (*ry*). *dl* dorsal lobe of brain, *pr* proboscis, *vl* ventral lobe of brain.

**Figure 2:**
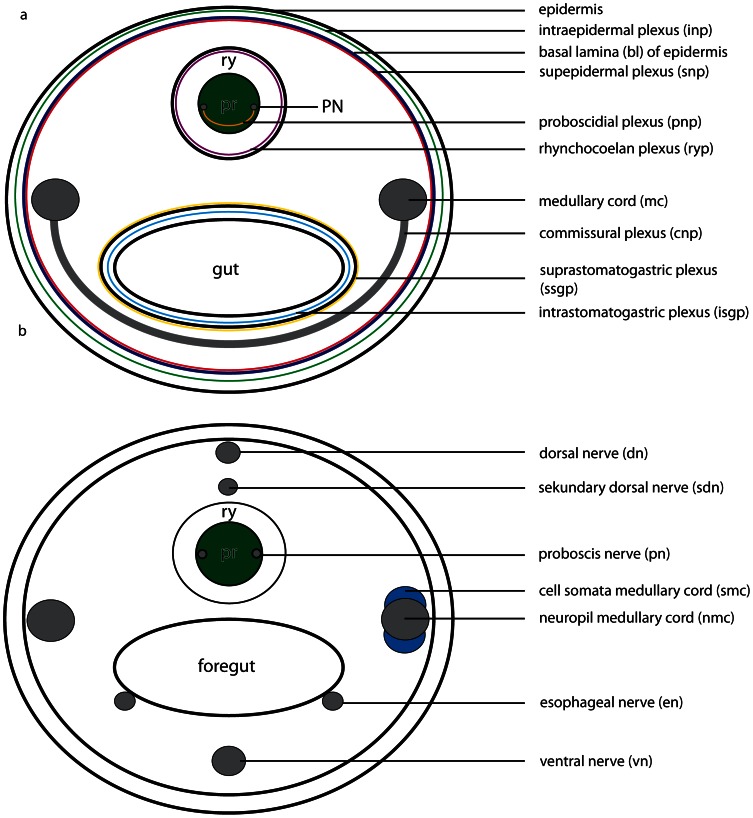
Schematic drawings of the peripheral nervous system in nemerteans. **a**: Scheme of the different nerve plexus in nemerteans. **b**: Scheme of the different major nerves present in nemerteans. Note the location of the neuronal cell somata (*blue*) of the medullary cords. The neuronal cell somata overlie the neuropil of the medullary cords dorsally and ventrally. *pr* proboscis, *ry* rhynchocoel.

The neuroanatomical nomenclature used in this paper largely follows the terminology provided in the review by Richter et al. [21]. In order to define the specific terms of nemertean neuroanatomy, some general remarks will be given prior to describing the nervous system in each of the different species studied (figs. 1& 2). The main components of the nemertean nervous system are the *central nervous system* consisting of a *brain* and *nerve cords*, the *peripheral nervous system* that includes *nerves* and *plexus*, and finally the *sensory structures*. While the *central nervous system* is characterized by a clear separation between neurites (*neuropil*) and their perikarya (*somata*), *nerves* are bundles of neurites with a few nuclei interspersed and are surrounded by extracellular matrix (*ecm*). *Plexus* are meshworks of neurites, and sensory organs are specialized, larger aggregations of several sensory cells.

### Central nervous system

#### Brain

An outer neurilemma of *ecm* may surround the entire central nervous system. The *somata* are located peripherally and externally to the central *neuropil*; *somata* and *neuropil* may be separated by an *inner neurilemma* consisting of *ecm* (fig. 1b). The *brain* is always bilaterally symmetrical and may contain subunits, or compartments like *lobes* which connect contralaterally by *commissural tracts* and ipsilaterally by *lateral tracts*.

#### Nerve cords

*Nerve cords* are locally-restricted, longitudinal strands of *neuropil* surrounded by the somata of their neurons. A pair of *medullary cords* is ventro-laterally located. They extend throughout the body and originate from the posterior ventro-lateral margin the brain. The distribution of the *somata* relative to the *medullary cord neuropil* varies among the taxa (fig. 2b). *Cephalic nerve cords* originate from the anterior margin of the brain.

#### Neurons

The *somata* are grouped peripherally to the neuropil and differ in their morphology and staining affinities. According to the structure of their perikarya (somata), four different size classes have been established [4], [27]. Somata of class 1 neurons (*S1*) are small and often have a variable shape, while somata of class 2 neurons (*S2*) are spherical to pear-shaped and are twice as large as *S1*, and somata of class 3 neurons (*S3*) are spherical to drop-shaped with large nuclei, a prominent nucleolus and are twice as large as *S2*. Class 4 cells are known as *neurochord cells*, but it is unknown whether these are neurons at all.

### Peripheral nervous system and sensory structures

The nemertean peripheral nervous system consists of several minor nerves, such as the *cephalic nerves*, the *proboscis nerves*, *dorsal* and *ventral nerves*, *esophageal nerves* and *nerve plexus* (figs. 1& 2).

#### Nerves

The *cephalic nerves* originate at the anterior margin of the brain and run towards the anterior tip of the animal. The *ventral nerve* is a single, median nerve that originates from the posterior margin of the *ventral commissural tract* and initially runs ventrally to the rhynchocoel and then more posteriorly, ventral to the gut. The *dorsal nerve* is a single, median nerve located dorsal to the rhynchocoel. It originates from the posterior margin of the *dorsal commissural tract* and runs posteriorly. A *second dorsal nerve* may be located ventral to the *dorsal nerve*. It originates from the dorsal nerve and runs posteriorly, directly above the rhynchocoel. Bundles of neurites regularly connect both nerves. A pair of *esophageal nerves* originates from the caudal margin of the *ventral commissural tract* or from the ventral lobes. Both nerves are commissurized anterior to the mouth opening, encircle the foregut, turn ventro-laterally and run posteriorly on either side of the gut (figs. 1a, 2b). In basal nemerteans the *proboscis nerves* originate from the dorsal margin of the *ventral commissural tract* and run posteriorly inside the proboscis wall (fig. 1a). The *proboscis nerves* are always paired, and may be multiplied in certain nemertean species.

#### Plexus

Both *medullary cords* may be interconnected by *commissural plexus* (fig. 2a). Other plexus are inside the proboscis wall (*proboscidial plexus*), in the wall of the rhynchocoel (*rhynchocoelan plexus*), in the gut epithelium (*intrastomatogastric plexus*), surrounding the gut musculature (*suprastomatogastric plexus*), underneath the basal lamina (*subepidermal plexus*) and inside the epidermis (*intraepidermal plexus*) (fig. 2a).

#### Sensory structures

Nemerteans possess several anterior *sensory organs*, such as the *frontal organ*, *eyes*, and *cerebral organs* (fig. 1a). The *eyes* are visible by their black pigment, whereas *cerebral* and *frontal organs* are defined by their morphology and position. *Frontal organs* consist of one to three densely ciliated pits located on the anterior tip of the head. *Cerebral organs* are tubular epidermal invaginations with distinct morphological differences among the lining cells and a close nervous connection to the brain. If present, they are always posterior to the brain in palaeonemertean species. Beside the sensory organs, isolated *sensory cells* are distributed across the entire body surface.

### *Procephalothrix filiformis* and *Cephalothrix linearis* (Cephalothricidae)

**Figure 3:**
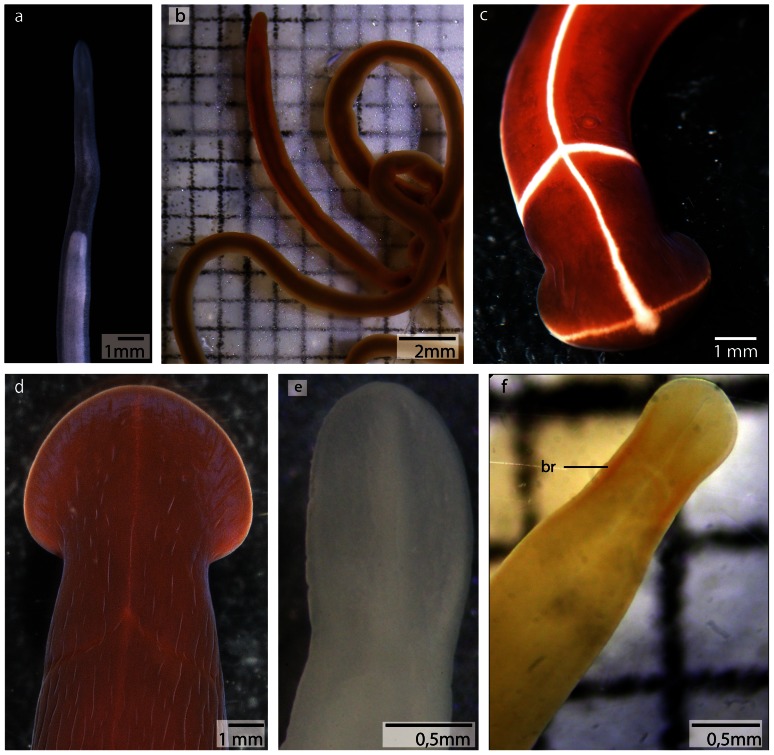
Living specimens of a: *Procephalothrix filiformis*. **b**: *Cephalothrix linearis*. **c**: *Tubulanus superbus*. **d**: *Tubulanus polymorphus*. **e**: *Callinera grandis*. **f**: *Carinina ochracea.* *br* brain.

**Figure 4:**
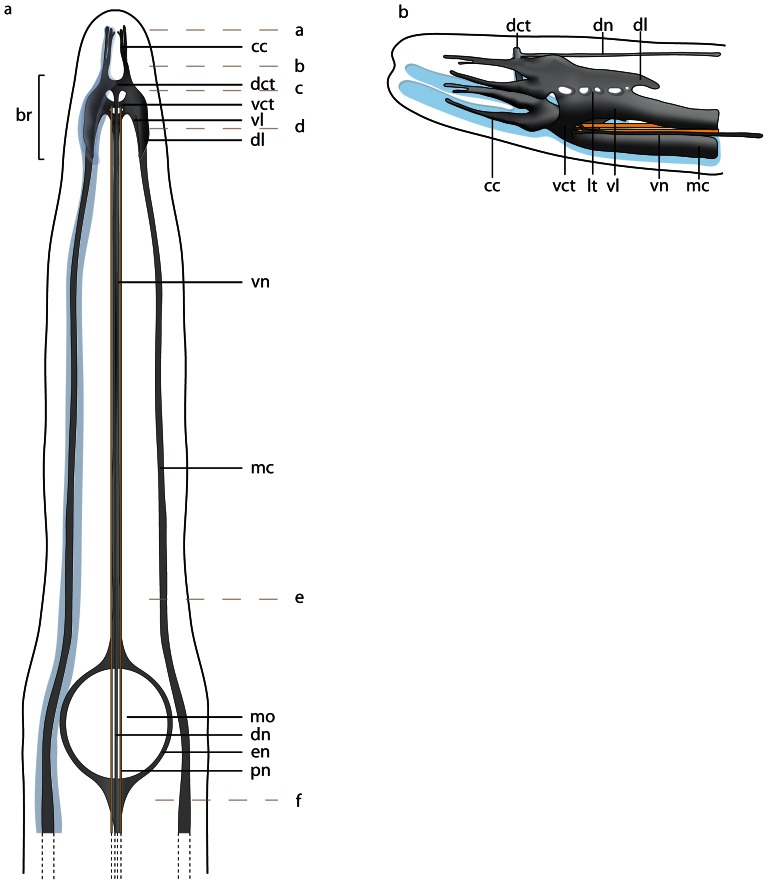
*Procephalothrix filiformis*, schematic drawings of the central nervous system based on 3D-reconstruction of 501 aligned 0.5 µm sections, dorsal (a) and lateral (b) view. **a**: The nervous system is composed of neuropil (*np*, *gray*) which may be surrounded by cell somata (*cs*, *blue*). Cephalic cords (*cc*) are circular arranged around the head of the animals. The paired proboscidial nerves (*pn*, *yellow*) originate from the ventral commissural tract (*vct*). A dorsal nerve (*dn*) originates from the dorsal commissural tract (*dct*), a ventral nerve (*vn*) from the ventral commissural tract. The branching esophageal nerves (*en*) originate from the ventral nerve and surround the mouth opening (*mo*). The lateral medullary cords (*mc*) originate in the ventral lobes of the brain (*br*). Letters on the right (**a**–**f**) refer to the histological sections in figure **5**. The nerve plexus originating in the dorsal nerve (see fig. **5e**) is omitted. **b**: Central nervous system lateral view. The dorsal lobe (*dl*) of the brain and the ventral lobe (*vl*) of the brain are connected by several lateral tracts (*lt*).

*Cephalothricid* species are characterized by a long and bluntly pointed head [5] that is termed rostrum. Their brain is located in the rostrum of the animals, far anterior to the mouth opening (figs. 3a, b; 4), and embedded in the head musculature. The nervous system in the two species studied is almost identically organized (see https://www.morphdbase.de?P_Beckers_20130201-M-15.1 for *Procephalothrix filiformis* stack and https://www.morphdbase.de?P_Beckers_20130201-M-14.1 for *Cephalothrix linearis* stack). For this reason immunhistological methods were only applied to *P. filiformis*.

### Central nervous system

#### Brain

**Figure 5:**
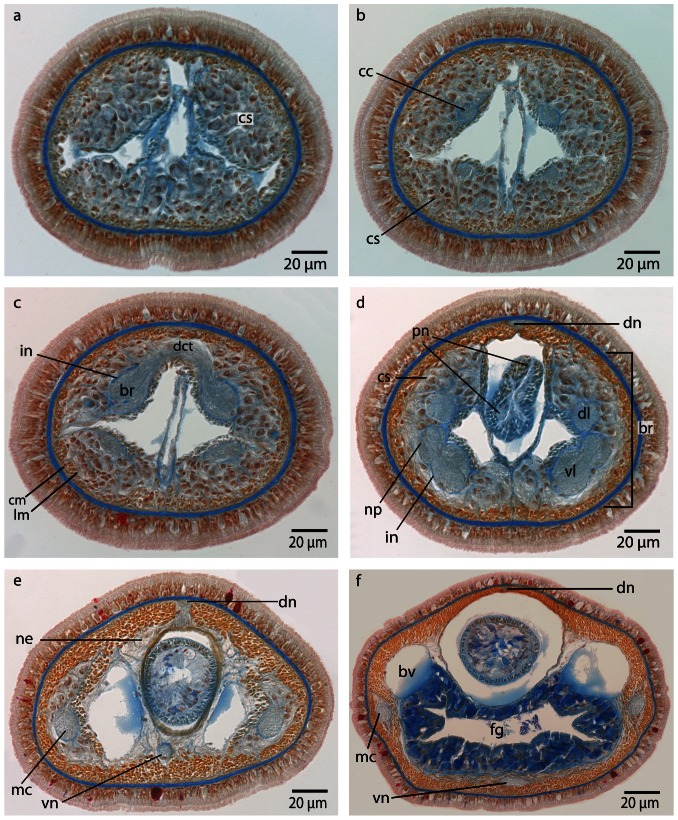
*Procephalothrix filiformis*, light micrographs of Azan stained transverse sections of the brain. **a**: Frontal part showing the huge layer of neuronal cell somata (*cs*). **b**: Four cephalic cords (*cc*) extend towards the tip of the head. **c**: A dorsal commissural tract (*dct*) connects the two halves of the brain (*br*). The neuronal cell somata are separated from the neuropil of the brain by an inner neurilemma (*in*). The brain is embedded in a layer of longitudinal muscles (*lm*), which lie underneath a layer of circular muscles (*cm*). **d**: The brain (*br*) is divided into dorsal (*dl*) and ventral (*vl*) lobes in its posterior part and composed of a central neuropil (*np*) which is surrounded by cell somata (*cs*). The somata are separated from the neuropil by an inner neurilemma (*in*), and two proboscidial nerves (*pn*) are present. **e**: A dorsal nerve (*dn*) arises from the dorsal commissural tract inside the musculature and migrates dorsally, a ventral nerve (*vn*) from the ventral commissural tract. Neurites (*ne*) of the dorsal nerve extends as a roof-like structure around the rhynchocoel. *mc*: medullary cord. **f**: The lateral medullary cords (*mc*) are embedded into the longitudinal muscle layer, and the dorsal nerve (*dn*) is located subepidermally. *bv* blood vessel, *fg* foregut, *vn* ventral nerve.

**Figure 6:**
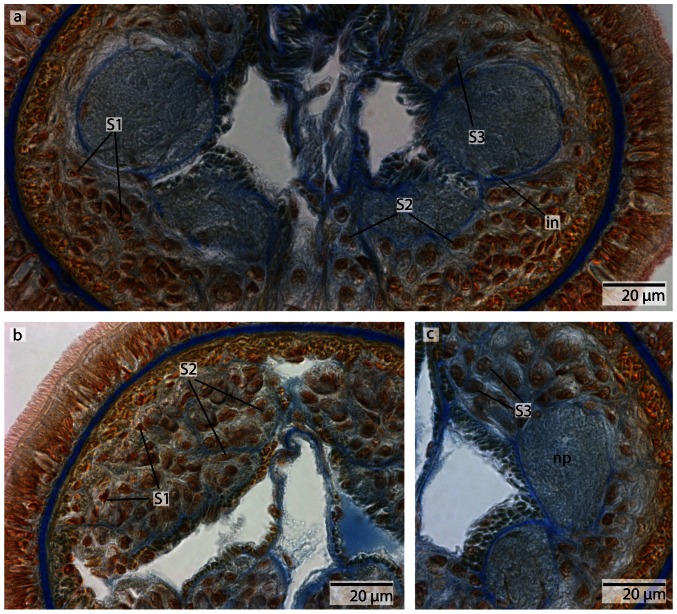
*Procephalothrix filiformis*, light micrographs of Azan stained neurons. **a**: Transverse section, overview of the brain, position of the three different types of neurons (*S1–S3*). **b**: Higher magnification of somata of type 1 and 2 neurons (*S1, S2*). Nuclei of S*1* dye orange and the cell bodies are beak shaped. Cell bodies of *S2* are circular and enlarged. Nuclei dye orange. **c**: The nuclei of cell somata type 3 (*S3*) also stain orange. The cell bodies are circular but more prominent than in *S1*–*S2*. *in* inner neurilemma, *np* neuropil.

**Figure 7:**
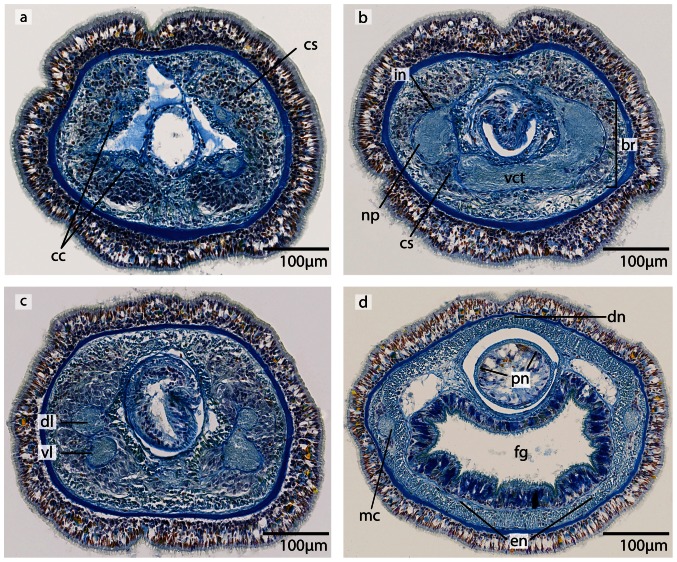
*Cephalothrix linearis*, light micrographs of Azan stained transverse sections of the brain. **a**: The 4 cephalic cords (*cc*) are frontally covered by an enormous layer of cell somata (*cs*). **b**: The brain (*br*) is composed of a central neuropil (*np*) surrounded by cell somata (*cs*). The somata are separated from the neuropil by an inner neurilemma (*in*). A ventral commissural tract (*vct*) connects the two parts of the brain. **c**: The posterior part of the brain is divided into dorsal (*dl*) and ventral (*vl*) lobes. **d**: the ventral lobes are confluent with the lateral medullary cords (*mc*), two proboscidial nerves (*pn*) and esophageal nerves (*en*) are present. A dorsal nerve (*dn*) extends to the posterior underneath the basal lamina of the epidermis. *fg* foregut.

**Figure 8:**
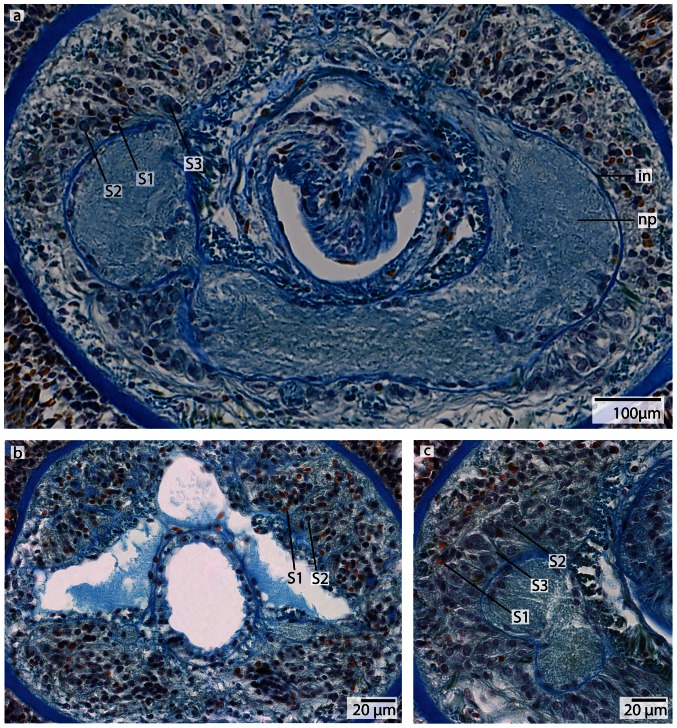
*Cephalothrix linearis*, light micrographs of Azan stained neurons. **a**: Transverse section, overview of the brain, position of the three different types of neurons (*S1*–*S3*). **b**: Frontally, only type *1* and type *2* neurons are present. **c**: Nuclei of type *1* neurons stain red. Nuclei of type *2* neurons stain bright purple; the cell body is slightly enlarged. The cell body of type *3* neurons is the most prominent. Their nuclei stain red. *in* inner neurilemma, *np* neuropil.

**Figure 9:**
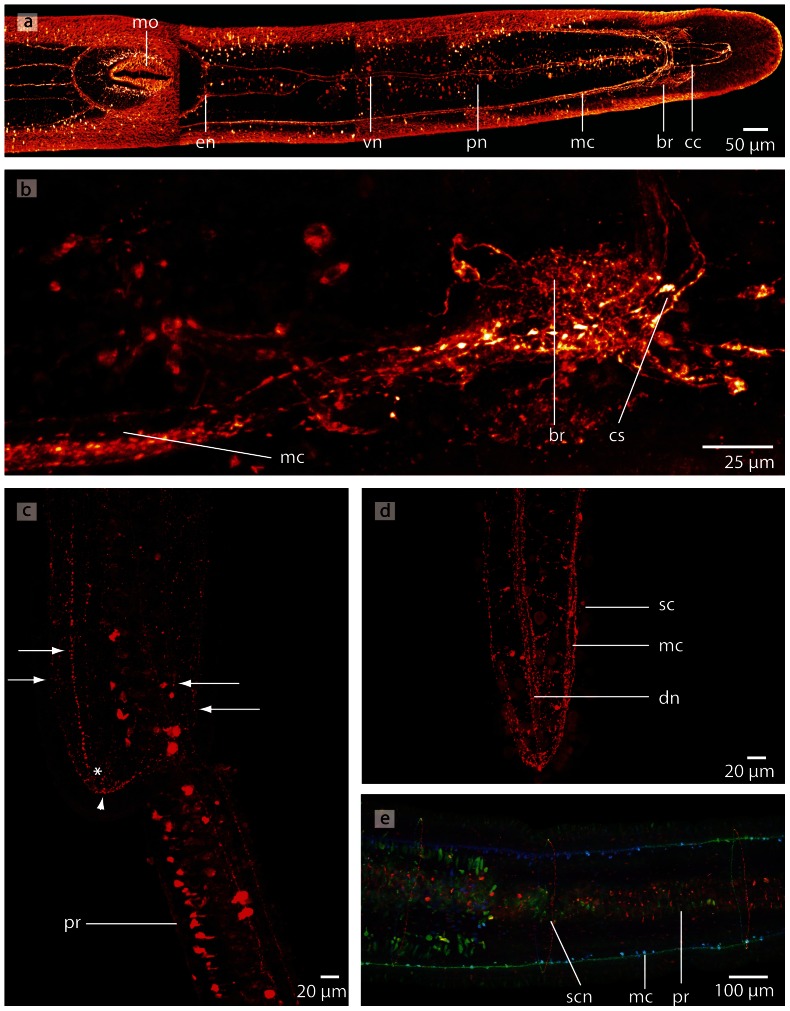
*Procephalothrix filiformis*, confocal laserscanning (cLSM) micrographs of differently immunostained whole mounts. **a**: Anti-FMRF. The cephalic cords (*cc*) originate in the lateral aspects of the brain (*br*). The lateral medullary cords (*mc*) extend the full length of the animal. There are two proboscidial nerves (*pn*) that run opposed to each other, along both sides of the proboscis. The esophageal nerves (*en*) originate at the ventral nerve (*vn*) and branch shortly before the mouth opening (*mo*). **b**: Anti-FMRF. Only few neuronal cell somata (*cs*) of the brain (*br*) are immunoreactive against FMRF. **c**: Anti-FMRF. Four minor nerves (*arrow*) unite in the tip of the animals' head (*arrowhead*). The nerves are interconnected by a circular nerve (*asterisk*) **d**: Anti-FMRF. The dorsal nerve (*dn*) is connected to the medullary cords (*mc*) in the very posterior part of the animal; bottle shaped sensory cells (*sc*) are distributed all over the body. **e**: Anti serotonin. The medullary cords (*mc*) are connected by serial arranged circular nerves (*scn*). Note that the proboscis nerves show no immunoreactivity against serotonin. *pr* proboscis.

The central part of the brain neuropil is separated from the neuronal somata by an inner neurilemma consisting of extracellular matrix (*ecm*), which was indicated by blue coloration in the Azan staining (figs. 5d, 6a; 7b, 8a). An outer neurilemma is not visible. The brain consists of four distinct, lobe-like compartments that anteriorly are juxtaposed to the head lacuna (figs. 5c,d; 7b,d). The dorsal lobes are located several micrometers anterior to the ventral lobes. A small dorsal commissural tract connects both dorsal lobes (fig. 5c). It originates at the anterior-dorsal margin of each lobe, next to the origin of the dorsal cephalic nerve cord. The dorsal lobes are more voluminous than the ventral ones. Each dorsal lobe is bean-shaped, and tapers posteriorly to form a small process that ends in a layer of neuronal cell somata. On each side of the brain, a series of at least five lateral tracts connects the dorsal lobe to the ventral lobe. When numbered along the anterior-posterior axis, the third lateral tract is the broadest of these tracts. The ventral lobes are connected by a prominent ventral commissural tract, which is located posterior to the dorsal commissural tract and originates at the anterior-ventral margin of each ventral lobe, next to the insertion of the first lateral tract (fig. 9b). The neurites from each commissural tract are horizontally arranged. Each ventral lobe also tapers posteriorly and is confluent with the medullary cords (figs. 5e; 9a; 7d).

#### Medullary cords

The medullary cords are ventro-laterally located and embedded into the longitudinal muscle layer. They travel along the animal to merge at its posterior end (figs. 9d). The neuropil of the medullary cords is capped by two crescent-shaped layers of neuronal somata, which form a dorsal and a ventral cap in transverse sections (fig. 5f; 7d). The neuronal cell somata are separated from the neuropil by an inner neurilemma. Some neurites, however, leave the neuropil and arborize between the cell somata (fig. 5e). The medullary cords are interconnected by serially arranged small circular neurites that show immunoreactivity against an antiserum directed against serotonin in *P. filiformis* (fig. 9e).

#### Cephalic nerve cords

In *P. filiformis* and *C. linearis*, a massive layer of neuronal somata is located frontal to the brain, underneath the outer circular and the inner longitudinal muscle layers (figs. 5a). The somata surround four strands of neuropil, and are seperated by an inner neurilemma (fig. 5b, 7a). In the literature they have been termed cephalic nerves [5]. According to the definitions above, however, these four strands are part of the central nervous system and must be termed cephalic nerve cords. These four nerve cords are arranged in pairs, a dorsal and ventral one, which are so tightly apposed to the head lacuna that their lumen seems to be compressed (fig. 5a). The dorsal cords are anteriorly bifurcated, while the ventral cords simply taper anteriorly. The dorsal cephalic nerve cords originate from the anterio-dorsal margin of the dorsal lobes, directly underneath the origin of the dorsal commissural tract. The ventral cephalic nerve cords originate from the anterio-ventral margin of the dorsal lobes, anterior to the first lateral tract that interconnects the dorsal and ventral lobes. The somata of the cephalic nerve cords in *P. filiformis* barely show any immunoreactivity against an antiserum directed against FMRFamid or serotonin (figs. 9a, b).

#### Neurons

In both cephalothricid species, the neurons can be classified according to their size, as proposed by Bürger [4], with class 2 neurons having somata and nuclei that are twice as large as class 1 neurons and somata and nuclei are half as large as class 3 neurons. All differ in structure, shape and Azan staining ability (figs. 6a–c, 8a–c). In *P. filiformis* cell bodies of class 1 neurons are slender and beaked with orange staining cell bodies and small, red nuclei that appear condensed (diameter: 0.92±0.15 µm, n = 12). These neurons are rare, but evenly distributed all over the brain (fig. 6a). Somata and nuclei of class 2 neurons are globular, having cell bodies almost twice as large as that of class 1 neurons (fig. 6b). Azan stains the cell body and nuclei of class 2 neurons bright to grayish orange. Class 2 neurons are the most prevalent cell type throughout the brain and the entire central nervous system (diameter: 1.85±0.21 µm, n = 11). Class 3 somata are globular, having a grayish Azan coloration, while the nuclei stain dark orange (diameter: 4.05±0.67 µm, n = 13). Class 3 neurons are evenly distributed throughout the brain, although they are less frequent than class 2 neurons (fig. 6c). All size classes differ significantly in diameter (p<0.001).

In *C. linearis*, class 1 neurons are small and have nuclei stained red (figs. 8a–c). The greatest accumulation of class 1 neurons is found in the cephalic nerve cords, although they are also found rarely but regularly distributed all over the brain (fig. 8a) (diameter: 2.86±0.41 µm, n = 10). Cell bodies of class 2 neurons are drop-shaped, stain bright purple with darker nuclei (figs. 8b, c), and are found primarily in the posterior ventral part of the brain, arranged in clusters (diameter: 5.38±0.86 µm, n = 11). The nucleus of class 3 neurons shows the same bright purple coloration as its soma (fig. 8c), but contains a large, red nucleolus (diameter: 10.68±1.49 µm, n = 13). Class 3 neurons are found in the middle of the brain, just behind the ventral commissural tract. In *P. filiformis* the majority of neurons belong to type 2 neurons, whereas in *C. linearis*, most neurons belong to type 1 and type 2. In the posterior part of the medullary cords, the layer of neuronal somata decreases in thickness.

Although Azan staining reveals an enormous layer of cell somata in the brain, staining *P. filiformis* with an antiserum against FMRFamid marks only very few cells (figs. 9a, b). The neuropil is not only restricted to the central part of the brain (clearly separated by *ecm* from the somata), but is also branching into the neuronal cell somata layer (fig. 5d).

### Minor nerves and peripheral nervous system

**Figure 10:**
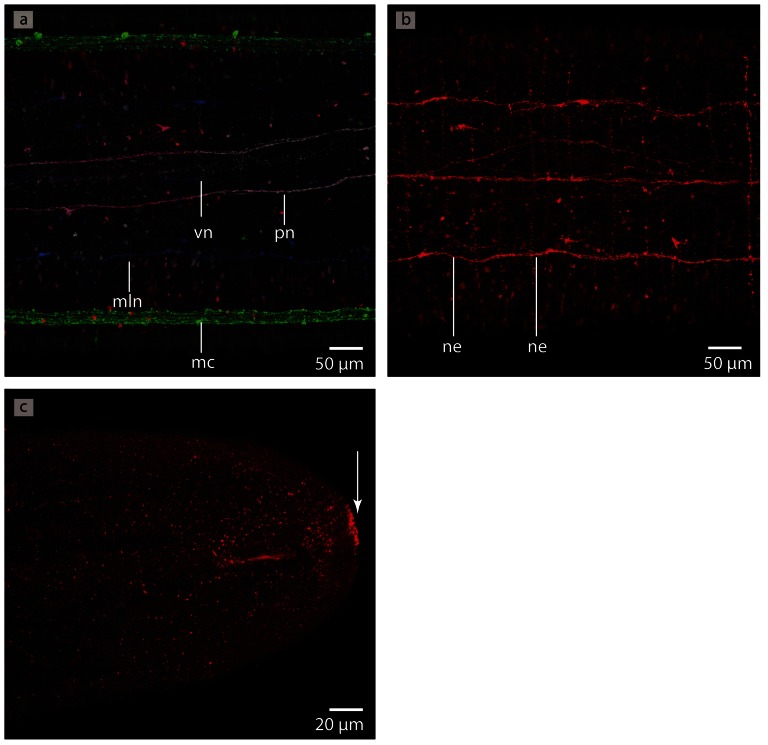
*Procephalothrix filiformis*, confocal laserscanning (cLSM) micrographs of differently immunostained whole mounts of. **a**: Anti-FMRF, z-coded stack. The major lateral medullary cords (*mc*), some minor lateral nerves (*mln*) as well as a ventral nerve (*vn*) are visible. *pn*: proboscis nerves. **b**: Anti-FMRF. The neurites (*ne*) of the intraepidermal plexus are arranged in a ladder-like way. **c**: Anti-FMRF. On the very tip of the head is a cluster of cells showing immunoreactivity against FMRF (*arrow*).

In the *P. filiformis* head, four minor nerves show immunoreactivity against an antiserum directed against FMRFamid and serotonin. These nerves extend from the tip of the head to the posterior end of the animal (figs. 9c; 10a). A circular commissural nerve connects these nerves (fig. 9c). In both cephalothricid species, a single **dorsal nerve** originates from the posterior margin of the dorsal commissural tract and runs posteriorly within the outer longitudinal muscle layer. More posteriorly, it passes the outer circular muscle layer to run underneath the basal lamina of the epidermis. The dorsal nerve then merges into the connection between the lateral medullary cords at the posterior end of the animal (figs. 5d–e; 7d). The **ventral nerve** has two roots, which branch off where the ventral lobes are connected by the ventral commissural tract and run medially for a few micrometers before fusing and running posteriorly ventral to the rhynchocoel. A few micrometers in front of the mouth, this nerve splits into two branches that surround the foregut. Both branches fuse behind the mouth and run posteriorly ventral to the gut as a single ventral nerve (figs. 4a; 5f; 9a). **Esophageal nerves** are lacking in both cephalothricid species. In *P. filiformis* staining against an antiserum directed against FMRFamid reveals two additional minor nerves, which originate behind the mouth (fig. 9a) and travel to the posterior of the animal. In both species, two **proboscidial nerves** arise from the posterior margin of the ventral commissural tract (fig. 4). The nerves run posteriorly on either side of the proboscis and are occasionally joined by a proboscidial plexus. In *P. filiformis*, the longitudinal proboscis nerves show immunoreactivity with an antiserum directed against FMRFamid, but not against serotonin (figs. 5d; 9c, e).

In *P. filiformis* a bright gray coloration in Azan stained sections clearly indicates a **nervous plexus** that arises from the dorsal nerve and forms a roof-like structure around the rhynchocoel (fig. 5e). Immunoreactivity with an antiserum directed against FMRFamid provides strong evidence that the neurites of the intraepidermal plexus are arranged in a regular, grid-like way (fig. 10b). A series of circular neurites connect the medullary cords (fig. 9e). The arrangement of the neurites of other plexus could not be reconstructed due to a lack of signal.

### Sensory structures

Although adult *P. filiformis* react strongly to changes in the illumination (photonegative) (unpubl. observations), there are no pigmented photosensory structures present. In the very tip of the head, a cluster of cell shows immunoreactivity with an antiserum directed against FMRFamid (fig. 10c). There are many cells distributed inside the epidermis and all over the body, which show immunoreactivity with an antiserum directed against FMRFamid and serotonin. The cells are bottle-shaped and may represent sensory cells (fig. 9d). A combination of immunostaining and electron microscopy should be employed to test this. Neither a frontal organ nor cerebral organs are present in either cephalothricid species.

### *Carinoma mutabilis* (Carinomidae, “Palaeonemertea”)

The nervous system of this species is located inside the longitudinal muscle layer; the brain lies anterior to the mouth; (see https://www.morphdbase.de?P_Beckers_20130201-M-10.1 for *C. mutabilis* stack).

### Central nervous system

#### Brain

**Figure 11:**
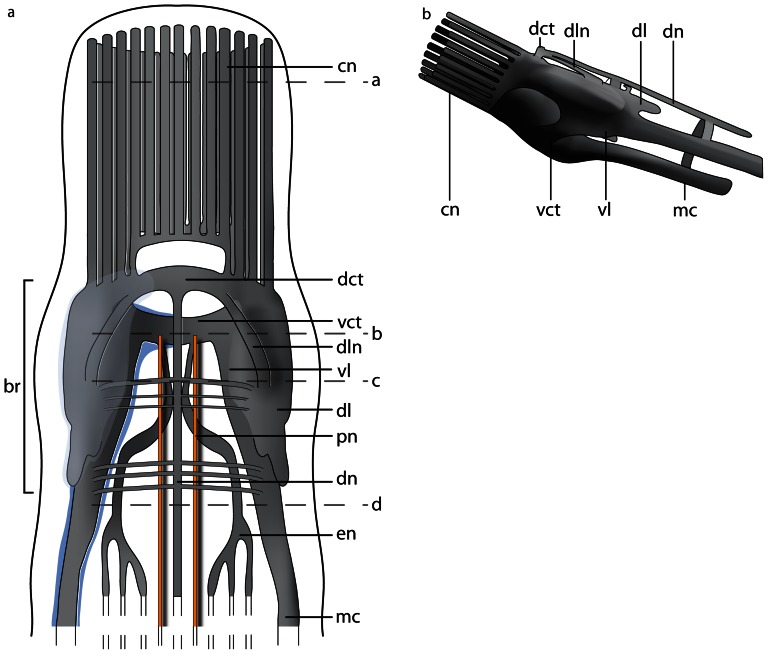
*Carinoma mutabilis*, schematic drawings of the central nervous system based on 3D-reconstruction of 137 aligned 0.5 µm sections, dorsal (a) and lateral (b) view. **a**: The nervous system is composed of neuropil (*np*, *gray*), which may be surrounded by cell somata (*cs*, *blue*). Cephalic nerves (*cn*) are circular arranged around the animal's head, and the paired proboscidial nerves (*pn*, *yellow*) originate from the ventral commissural tract (*vct*). The posterior part of the brain is divided into a dorsal (*dl*) and ventral lobe (*vl*). A dorso-lateral nerve (*dln*) connects the posterior part of the dorsal lobe with the dorsal commissural tract. A dorsal nerve strand (*dn*) originates from the dorsal commissural tract (*dct*). The branching esophageal nerves (*en*) originate from the ventral commissural tract. The lateral medullary cords (*mc*) originate in the ventral lobes of the brain. The letters on the right (**a**–**d**) refer to the histological sections in figure **12**. **b**: The cephalic nerves (*cn*) originate in the lateral parts of the brain. Only the posterior region of the brain is divided into a ventral (*vl*) and dorsal lobe (*dl*).

**Figure 12:**
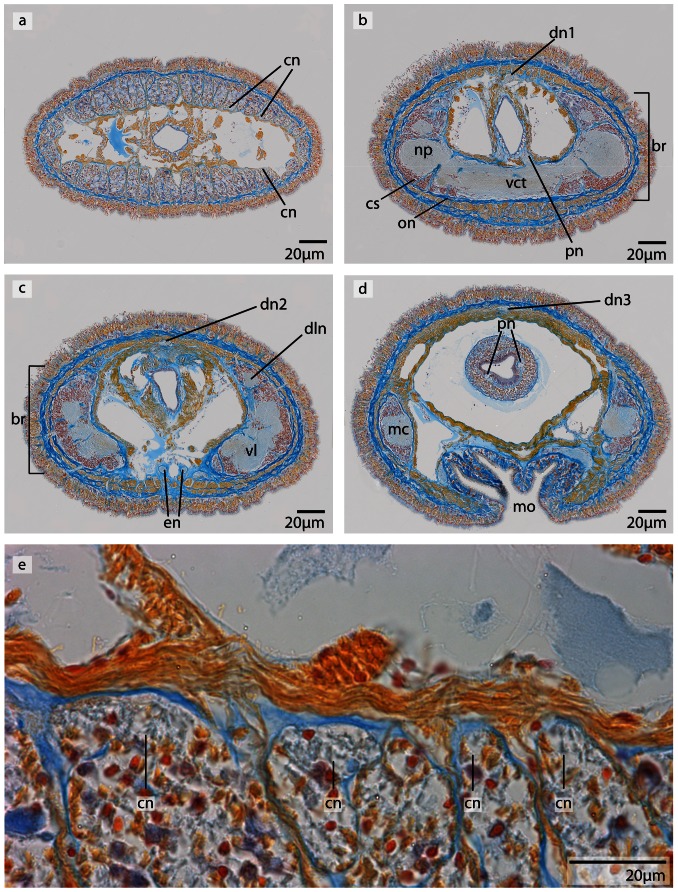
*Carinoma mutabilis*, light micrographs of Azan stained transverse sections of the brain. **a**: The cephalic nerves (*cn*) are circularly arranged around the inner margins of the head. **b**: The brain (*br*) is composed of a central neuropil (*np*) surrounded by cell somata (*cs*). The whole brain is encircled by an outer neurilemma (*on*). The two proboscidial nerves (*pn*) originate from the ventral commissural tract (*vct*). **c**: A dorsal nerve (*dn*) arises from the dorsal commissural tract, the esophageal nerves (*en*) originate from the ventral commissural tract, the brain is in its posterior part divided into a dorsal (*dl*) and ventral (*vl*) lobe. Dorso-lateral nerves (*dln*) arise from the dorsal commissural tract and merge with the dorsal lobes. d: The ventral lobes are confluent with the lateral medullary cords (*mc*), two proboscidial nerves are present (*pn*). Note the different positions of the dorsal nerve (*dn1–dn3*). e: Higher magnification of the anterior head region, showing the location of the cephalic nerves (*cn*). *dn* dorsal nerve, *mo* mouth opening.

**Figure 13:**
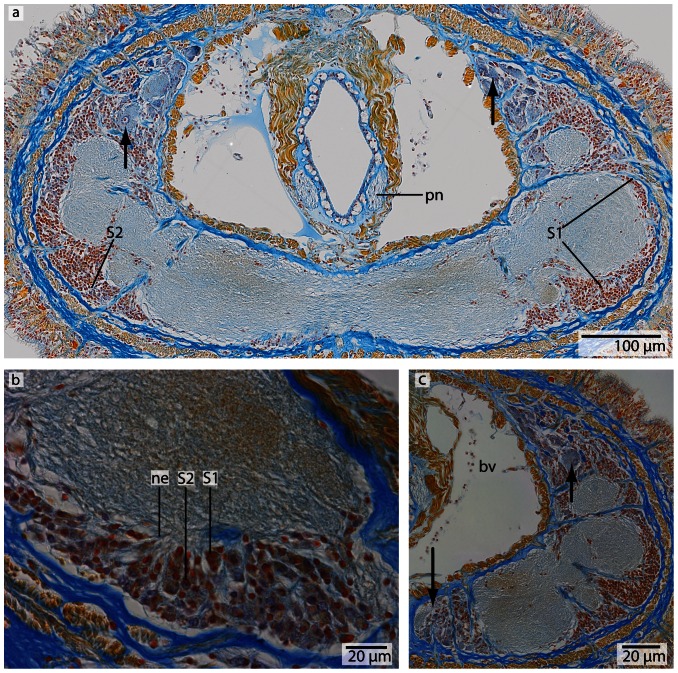
*Carinoma mutabilis*, light micrographs of Azan stained neurons. **a**: Transverse section, overview of the brain, position of the two different types of neurons (*S1*–*S2*). Another type of cells is located in the dorsal tip of the brain (*arrow*). **b**: Higher magnification of somata of type *2* neurons (*S2*). The nuclei of *S2* dye purple. The neurites (*ne*) of the brain neuropil branch into the somata layer. **c**: In the dorsal and ventral tip of the brain, cells with *red*-stained nuclei and very prominent cell bodies (*arrows*) are found. Note the close association of these cells with the blood vessels (*bv*). These cells resemble glandular cells. *pn* proboscis nerve.

The entire brain is surrounded by an outer neurilemma; an inner neurilemma is not present (figs. 11, 12b, c; 13a). The central neuropil of the brain is surrounded by a layer of neuronal cell somata (figs. 12b, c; 13a). The *Carinoma mutabilis* brain is situated anteriorly in front of the mouth opening. Like in the cephalothricid species, it is surrounded by the head musculature. The brain is not externally visible. It consists of paired dorsal and ventral lobes (fig. 12c). Three commissural tracts connect the dorsal lobes, the posterior two being small, closely associated and widely separated from the third prominent anterior dorsal commissural tract. The lateral portion of the anterior dorsal commissural tract and the anterior margin of the dorsal lobe give rise to cephalic nerves (figs. 11; 12a). The dorsal- and ventralmost of these nerves branch off to give rise to further cephalic nerves until a complete ring of more than thirty cephalic nerves is formed.They are located within the head musculature. The dorsal commissural tract also gives rise to a pair of posteriorly directed dorso-lateral nerves. On either side, they turn latero-ventrally and merge with the dorsal lobe where the second dorsal commissural tract originates (fig. 11). Directly posterior the third commissural tract, a horn-like extension projects from the inner margin of the dorsal lobe. Posterior to this structure, the dorsal lobe tapers to form a small process that ends within a cup of neuronal cell somata. The dorsal lobes of the brain are more voluminous than the ventral ones and are located anterior to them. The ventral lobe are located slightly ventro-median to them and slightly oblique to the main body axis. On either side the ventral lobe is broadly connected to the dorsal one, but several small bundles of *ecm* and occasionally muscle fibers, pierce this connection and cause the impression of several closely packed lateral tracts connecting both lobes (fig. 12b). Both ventral lobes are connected by a broad ventral commissural tract located between the origin of the first and the second dorsal commissural tract (fig. 12b). The neurites of the ventral commissural tract are horizontally arranged.

#### Medullary cords

The ventral lobes taper caudally and give rise to the lateral medullary cords. The medullary cords run posteriorly between the outer longitudinal muscle layer and the inner circular muscle layer. The neuronal cell somata of the medullary cords are located dorsally and ventrally to the neuropil (fig. 12d). The location of the medullary cords was observable until they entered the gonad region.

#### Neurons

The somata of the neurons fall into two size classes, with somata and nuclei of class 2 neurons being twice as large as those of class 1 neurons (figs. 13a, b). As the nuclei of class 1 neurons stain red, and a cytoplasmic surrounding is hard to detect, the cell somata of class 1 neurons must be very small (fig. 13b) (diameter: 1.75±0.32 µm, n = 12). These neurons are distributed all over the brain and the medullary cords, making them by far the dominant class of neurons. The cell somata of class 2 neurons stain purple and their nuclei are red (figs. 13a, b) (diameter: 5.26±0.72 µm, n = 10). These neurons are less frequently found in the central nervous system. The two size classes differ significantly in diameter (p<0.001).

A third type of cell is restricted to the dorso-median region of the dorsal lobe between the first and the second dorsal commissural tracts. There is also a ventro-median patch of these cells at the posterior margin of the ventral commissural tract and the origin of the esophageal nerves. The bodies of these cells are at least ten times more voluminous as those of the neurons, and contain nuclei at least four times larger. These cells possess a very prominent orange staining nucleolus. Dorsally these cells are in close contact with blood vessels, so they may represent glandular cells rather then neurons (figs. 13a, c).

### Minor nerves and peripheral nervous system

The **cephalic nerves** form a complete ring of longitudinal neurites with occasionally interspersed nuclei. Close to their origin, these nerves are interconnected, but are increasingly isolated from each other by an *ecm* as they run anteriorly. More than 35 of these nerves can be seen in transverse sections of the head region (fig. 12a). A **dorsal nerve** originates from the first dorsal commissural tract. Anteriorly the dorsal nerve is located in the musculature, but turns dorsally to extend beneath the epidermal basal lamina to the posterior end of the animal (figs. 12b–d). Very fine rings of neurites occasionally connect the dorsal nerve and the medullary cords. A **ventral nerve** is not present. The **esophageal nerves** arise from the posterior margin of the ventral commissural tract, turn medially and merge a few micrometers in front of the mouth. Posterior to the point of merging they bifurcate and surround the mouth on either side (fig. 12c). Posterior to the mouth, both nerves have two branches, all six of theserunning parallel to the esophagus. The paired **proboscidial nerves** originate from the anterio-dorsal margin of the ventral commissural tract and run posteriorly on either side of the proboscis (fig. 12d). Both are occasionally connected by a circular bundle of neurites.

### Sensory structures

No sensory structures such as eyes or cerebral organs are found in *C. mutabilis*. At the tip of the head, a medio-dorsal series of pits and slits with prominent sensory cilia and underlying neurons may represent the frontal organ. The regionally restricted large cells found in the dorso-median portion of the brain (figs. 13a, c) may represent glandular cells and might have a neurosecretory function.

### *Tubulanus superbus* and *Tubulanus polymorphus* (Tubulanidae, “Palaeonemertea”)

*Tubulanus* species are characterized by a sharply demarcated head [5], (figs. 3c, d). The anterior nervous systems of *Tubulanus superbus* and *Tubulanus polymorphus* is almost identically organized, although both species differ in size (see: https://www.morphdbase.de?P_Beckers_20130201-M-9.1 for *Tubulanus superbus* stack, https://www.morphdbase.de?P_Beckers_20130201-M-11.1 for *Tubulanus polymorphus* stack).

For this reason immunhistological methods are only shown for *T. polymorphus*.

### Central nervous system

#### Brain

**Figure 14:**
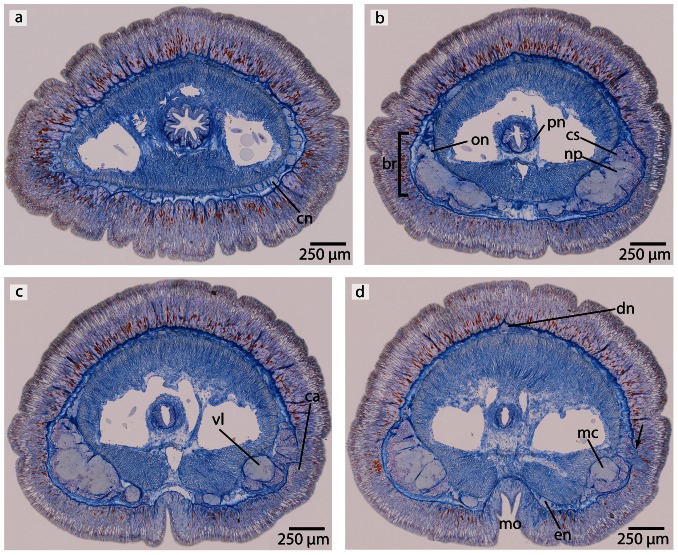
*Tubulanus superbus*, light micrographs of Azan stained transverse sections of the brain. **a**: The cephalic nerves (*cn*) are circularly arranged around the margins of the head. **b**: The brain is composed of a central neuropil (*np*) and surrounding cell somata (*cs*). An outer neurilemma (*on*) surrounds the whole brain. The two proboscidial nerves (*pn*) originate in the ventral commissural tract. **c**: Posteriorly the brain is divided into a dorsal and ventral lobe (*vl*). The canal (*ca*) of the cerebral organ does not pierce the basal lamina of the epidermis. **d**: The canal ends in a layer of neuronal cell somata (*arrow*) which have contact to the brain. Two esophageal nerves (*en*) are present. The dorsal nerve (*dn*) originates in a dorsal commissural tract. The ventral lobes of the brain are confluent with the lateral medullary cords (*mc*). *mo*: mouth opening.

**Figure 15:**
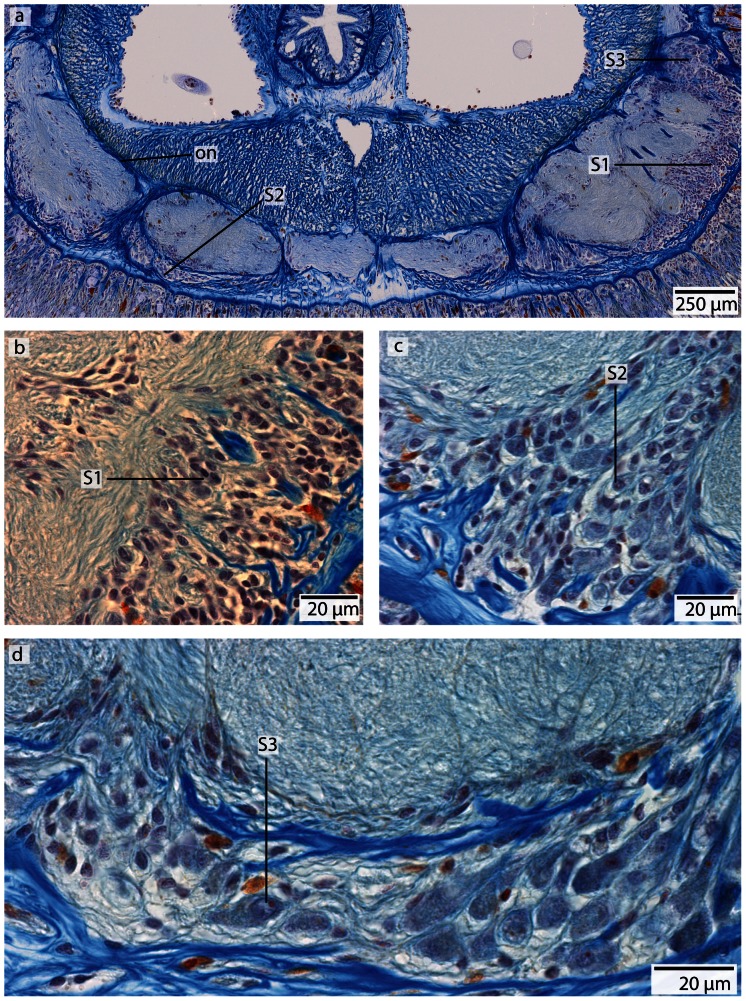
*Tubulanus superbus*, light micrographs of Azan stained neurons. **a**: Transverse section, overview of the brain, position of the different neuronal cell somata (*S1*–*S3*). **b**: The nuclei of type 1 neurons stain purple. The perikarya are not enlarged. **c**: Somata of type 2 neurons exhibit orange-stained nuclei. The perikarya are enlarged. **d**: Cell bodies of type *3* neurons are very prominent; the nuclei stain orange. *on* outer neurilemma.

**Figure 16:**
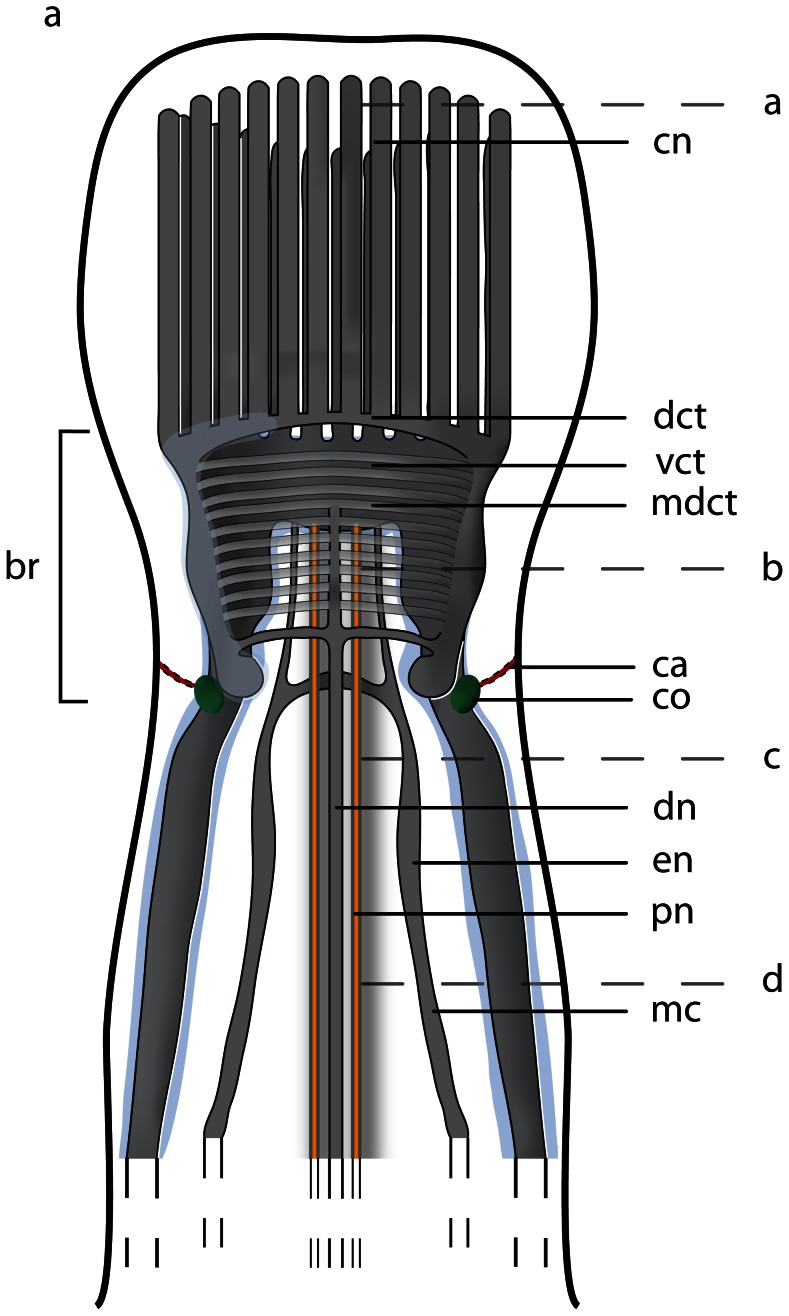
*Tubulanus polymorphus*, drawing of a dorsal view on the central nervous system, based on 3D-reconstruction of 173 aligned 0.5 µm sections. The nervous system is composed of neuropil (*np*, *gray*) which may be surrounded by cell somata (*cs*, *blue*). Cephalic nerves (*cn*) are circularly arranged around the animal's head, and the paired proboscidial (*pn*, *yellow*) nerves originate from the ventral commissural tract (*vct*). A dorsal nerve (*dn*) originates from the dorsal commissural tract (*dct*). The cerebral organs (*co*, *green*) contact to the environment via small canals (*ca*) which open laterally. The cerebral organ is connected to the dorsal lobe of the brain. The branching esophageal nerves (*en*) originate from the ventral commissural tract. The lateral medullary cords (*mc*) originate in the ventral lobes of the brain. The letters on the right (**a**–**d**) refer to the histological sections in figure **17**.

**Figure 17:**
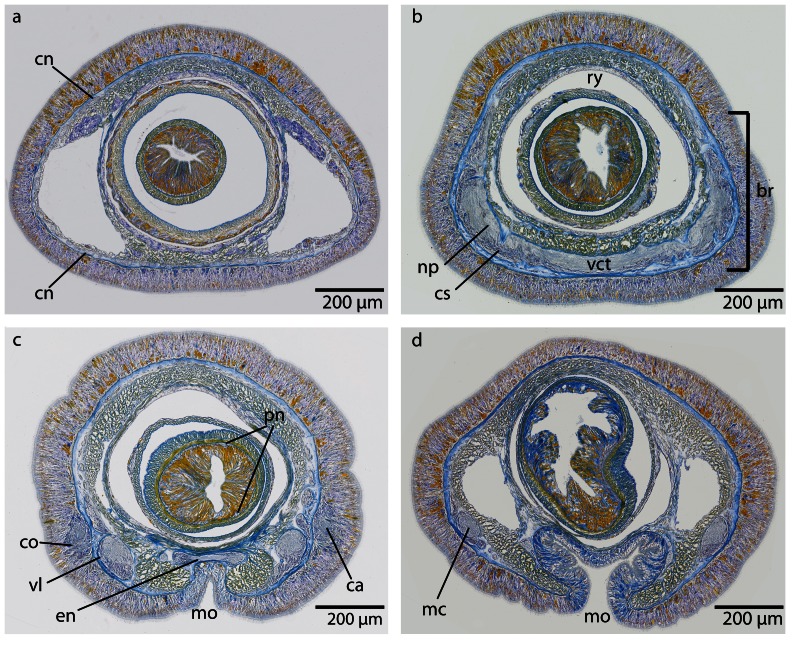
*Tubulanus polymorphus*, light micrographs of Azan stained transverse sections of the brain. **a**: The cephalic nerves (*cn*) are circularly arranged around the tip of the head. **b**: The brain (*br*) is composed of a central neuropil (*np*) which is surrounded by cell somata (*cs*), the ventral commissural tract (*vct*) connects the two halves of the brain below the rhynchocoel (*ry*). **c**: The canals (*ca*) of the cerebral organ (*co*) open ventro- laterally to the environment. The esophageal nerves (en) originate in the ventral commissural tract and are connected again shortly in front of the mouth opening (*mo*). Two proboscidial nerves (*pn*) are present. **d**: The ventral lobes of the brain are confluent with the lateral medullary cords. *mo* mouth opening, *vl* ventral lobes of brain.

**Figure 18:**
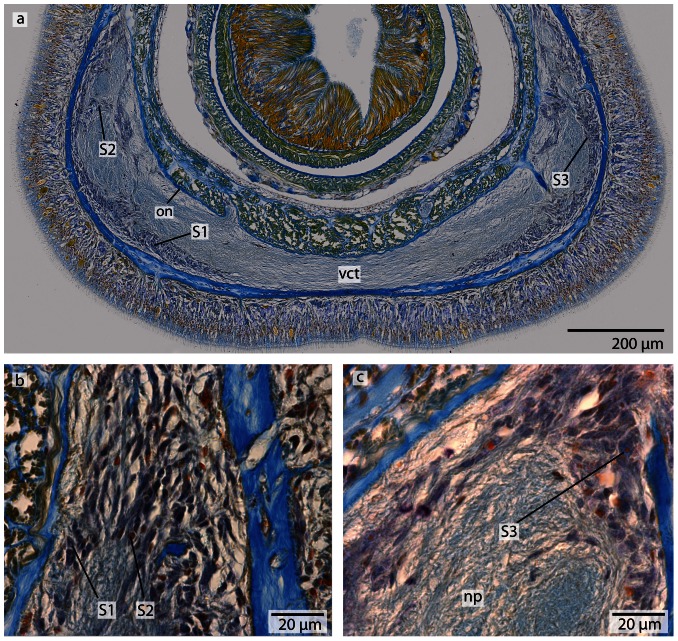
*Tubulanus polymorphus*, light micrographs of Azan stained neurons. **a**: Transverse section, overview of the brain; position of the three different types of neurons (*S1*–*S3*). **b**: Higher magnification of the somata of neuron types 1 and 2. Nuclei of type 1 neurons (*S1*) stain purple; the cell body is not enlarged. Nuclei of *S2* stain red. Type 2 neurons are solitarily distributed all over the brain. **c**: Nuclei of type *3* neurons stain red and the cell body is more prominent compared to *S1* and *S2*. *np* neuropil, *on* outer neurilemma, *vct* ventral commissural tract.

The brain of both *Tubulanus* species is composed of a central neuropil, which is surrounded by neuronal cell somata; a prominent outer neurilemma is present, while an inner neurilemma is missing (figs. 14b; 15a). In both species the brain is located ventro-laterally and anterior to the mouth, but is not externally visible (figs. 3d, e). The brain consists of four lobes that are located directly underneath the epidermal basal lamina (figs. 14a, 15b). The dorsal lobes are larger than the ventral lobes. They are connected by a series of dorsal commissural tracts, of which the anteriormost and the posteriormost are larger than the remaining ones (fig. 16). On either side of the brain, the dorsal and ventral lobes are connected by a continuous layer of neurites, which has a slightly smaller diameter than either of the lobes, making the lobes hard to distinguish (figs. 17b, 18a). Each connection represents a single broad lateral tract. It is, however, restricted to the region anterior to the posteriormost dorsal commissural tract. Each dorsal lobe has a posterior extension that does not connect to the ventral part of the brain and terminates in a layer of neuronal somata. Both ventral lobes are connected by a broad and prominent ventral commissural tract that lies posterior to the anteriormost dorsal commissures (figs. 14b; 16; 17b). The neurites of the ventral commissural tract are horizontally arranged (fig. 18a).

#### Medullary cords

Each ventral lobe is confluent with a lateral medullary cord, which runs posteriorly between the subepidermal basal lamina and the outer circular muscle layer (figs. 14d; 17d). In *T. superbus*, the neuronal somata of the medullary cords form an inverted “C” to cover the lateral, dorsal and ventral aspect of the neuropil (fig. 14d). Bundles of neurites branch into the layer of somata. In *T. polymorphus*, the neuronal somata of the medullary cords form two caps, which dorsally and ventrally cover the neuropil (fig. 17d). In this species, bundles of neurites branch into the layer of somata like in *T. superbus*.

#### Neurons

In both species, the neurons of the central nervous system fall into three size classes (figs. 15, 18). Like the cephalothricid species, somata and nuclei of class 2 neurons are twice as large as those of class 1 neurons and half as large as those of class 3 neurons. In *T. superbus*, the nuclei of class 1 neurons stain dark purple, are arranged in clusters and have small, elongated cell bodies (figs. 15a, b) (diameter: 2.90±0.44 µm, n = 14). The nuclei of class 2 neurons stain purple and their cell bodies are pyriform (figs. 15a, c). These neurons are less frequent than class 1 neurons, and occur evenly all over the brain (diameter: 5.56±0.72 µm, n = 13). The nuclei of class 3 neurons also stain purple and contain an orange staining nucleolus (figs. 15a, d) (diameter: 12.40±2.04 µm, n = 11). These neurons are only found in small numbers. The prevailing neuron type in the medullary cords is class 1 neurons.

In *T. polymorphus*, the nuclei of class 1 neurons stain dark purple, their cell bodies are small, elongated and appear in clusters (figs. 18a, b) (diameter: 2.29±0.56 µm, n = 13). The nuclei of class 2 neurons stain orange and their cell bodies have a circular appearance (figs. 18a, b) (diameter: 4.36±0.45 µm, n = 10). These cells are found in small numbers only, but are evenly distributed all over the brain. Cell bodies of class 3 neurons are 50% larger than those of class 2 neurons, having nuclei that are much larger than nuclei of the former two neuron types and stain bluish purple with a prominent orange nucleolus (figs. 18a, c). The prevailing neurons in the medullary cords are class 1 neurons. All size classes differ significantly in diameter (p<0.001).

### Minor nerves and peripheral nervous system

In both species **cephalic nerves** originate from the anterior margin of the ventral and the anteriormost dorsal commissural tract as well as from the ventral and dorsal lobes. While running to the anterior they form a complete ring, between the basal lamina and a layer of longitudinal muscles (figs. 14a, 16, 17a). An *ecm* (neurilemma) separates each nerve from its neighbor. The cephalic nerves are not covered by neuronal cell somata. The **dorsal nerve** of *T. superbus* originates in one of the small dorsal commissural tracts and runs posteriorly underneath the basal lamina of the epidermis (fig. 14d). In *T. polymorphus*, this nerve takes the same course, but arises from the posterior margin of the posteriormost dorsal commissural tract. A **ventral nerve** is not present. In *T. superbus*, two **esophageal nerves** (fig. 14d) originate from the posterior margin of the ventral commissural tract, although in *T. polymorphus* these nerves branch off the ventral lobes next to the origin of the ventral commissural tract. In both species, they run posteriorly, pass the mouth and follow the course of the foregut running on either of its sides. Anterior to the mouth, both nerves are connected by a commissure (figs. 16; 17c). In both species, two **proboscis nerves** arise from the ventral commissural tract more central to the origin of the esophageal nerves. The proboscis nerves run opposite to each other along both sides of the proboscis and are occasionally connected by a proboscidial plexus. The proboscis nerves arise from the ventral commissural tract. (figs. 14b; 16; 17c).

**Figure 19:**
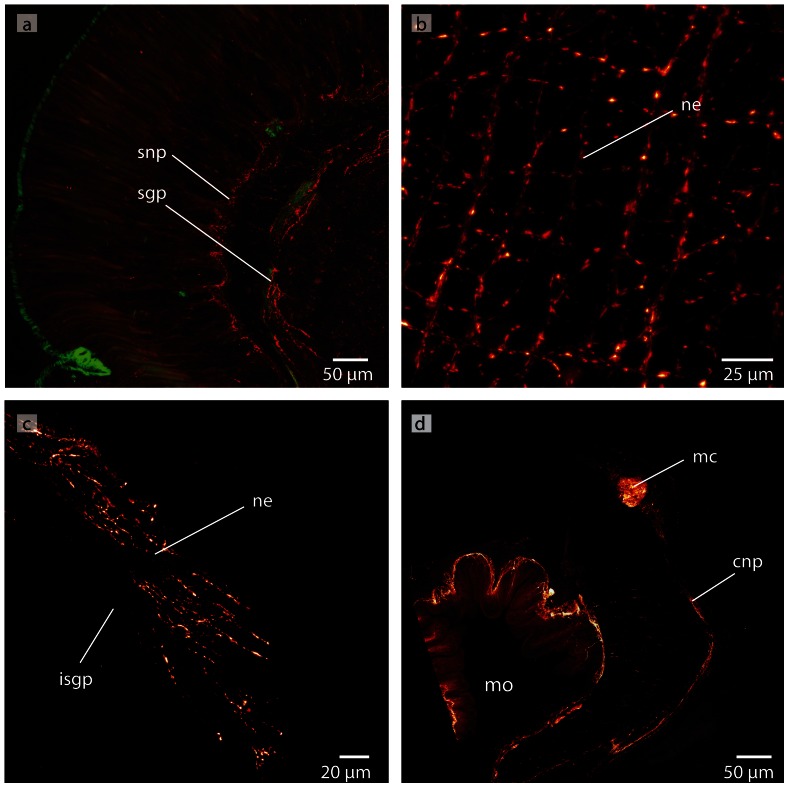
*Tubulanus polymorphus*, confocal laserscanning (cLSM) micrographs of differently immunostained vibratome sections. **a**: Transverse section, anti-FMRF (*red*), anti-α-tubulin (*green*) showing the subepidermal (*snp*) and the stomatogastric (*sgp*) nerve plexus. **b**: Horizontal section, anti-FMRF showing the regular arranged neurites (*ne*) of the subepidermal nerve plexus. **c**: Horizontal section, anti-FMRF. The neurites (*ne*) of the intrastomatogastric nerve plexus (*isgp*) are arranged in a regular ladder-like fashion. d: Transverse section, anti-serotonin. The lateral medullary cords (*mc*) are interconnected by a commissural plexus (*cnp*). The commissural plexus surrounds the mouth opening (*mo*).

Immunostaining reveals several **nerve plexus** in *T. polymorphus* (figs. 19a–d). The neurites of the intraepidermal plexus form a regular, ladder-like meshwork (fig. 19b). The neurites of the subepidermal plexus show immunoreactivity with an antiserum directed against FMRFamid (fig. 19a). The commissural plexus is well developed (fig. 19d) and connects the two medullary cords. The neurites of the latter two plexus are horizontally arranged. The neurites of the intrastomatogastric nerve plexus are arranged in an irregular net-like manner (fig. 19c).

### Sensory structures

#### Cerebral organ

**Figure 20:**
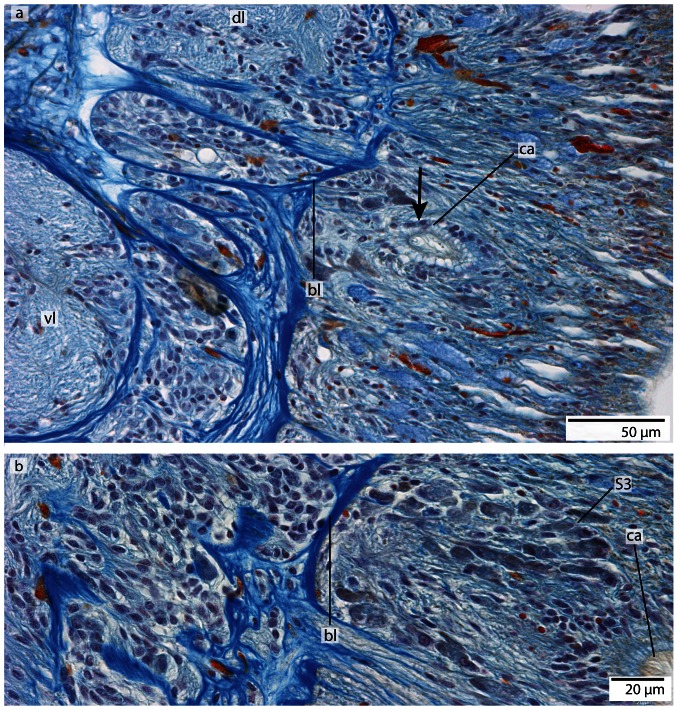
*Tubulanus superbus*, light micrographs of Azan stained transverse sections of the brain and cerebral organ. **a**: The sensory cells (*arrow*) surrounding the canal (*ca*) of the cerebral organ are connected to the dorsal lobe (*dl*) of the brain. The canal ends anterior to the basal lamina (*bl*) of the epidermis. **b**: The cells adjacent to the cerebral organ are type 1 brain cells. *vl*: ventral lobe.

Both *Tubulanus* species possess a pair of cerebral sense organs located in the mid-posterior part of the brain (fig. 16). Each cerebral organ consists of a simple ciliated tube that invaginates from a lateral epidermal pore (fig. 20a). The tube-like duct ends next to the posterior margin of the dorsal lobe. Some of the somata next to the duct belong to type 3 neurons in *T. superbus* and type 1 neurons in *T. polymorphus*, indicating that some of the ciliated cells lining the tube are sensory (fig. 20b). The inner section of the cerebral organ is connected to the dorsal lobe of the brain by small branches of neurites (fig. 20b). In both species there is no contact to the blood vessels.

### *Callinera grandis* (Tubulanidae, “Palaeonemertea”)

*Callinera grandis* has a subepidermal nervous system; the brain is located anterior to the mouth opening and is not visible from the exterior (fig. 3e), (see: https://www.morphdbase.de?P_Beckers_20130201-M-12.1 for *Callinera grandis* stack).

### Central nervous system

#### Brain

**Figure 21:**
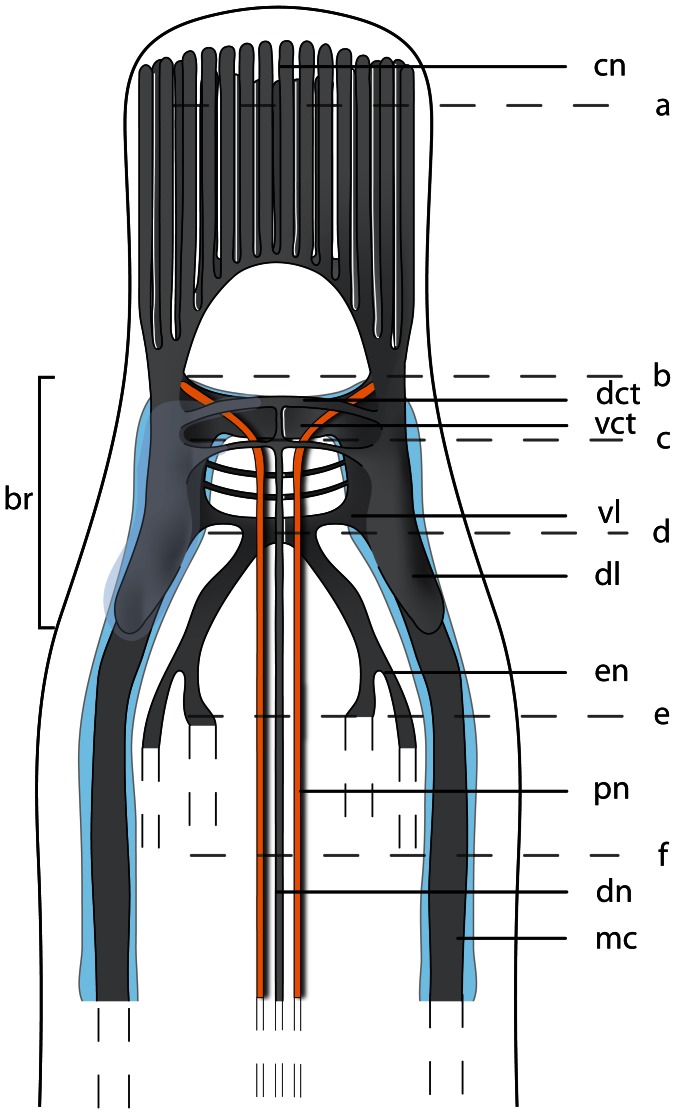
*Callinera grandis*, schematic drawing of a dorsal view on the central nervous system, based on 3D-reconstruction of 140 aligned 0.5 µm sections. The central nervous system is composed of neuropil (*np*, *gray*), which is surrounded by cell somata (*cs*, *blue*). Cephalic nerves (*cn*) are circularly arranged; the paired proboscidial nerves (*pn*, *yellow*) originate from the ventral commissural tract (*vct*). A dorsal nerve strand (*dn*) originates from the dorsal commissural tract. The branching esophageal nerves (*en*) originate from the ventral commissural tract. The lateral medullary cords (*mc*) originate ventro-caudally in the brain. The letters on the right (**a**–**f**) refer to the histological sections in figure **22**.

**Figure 22:**
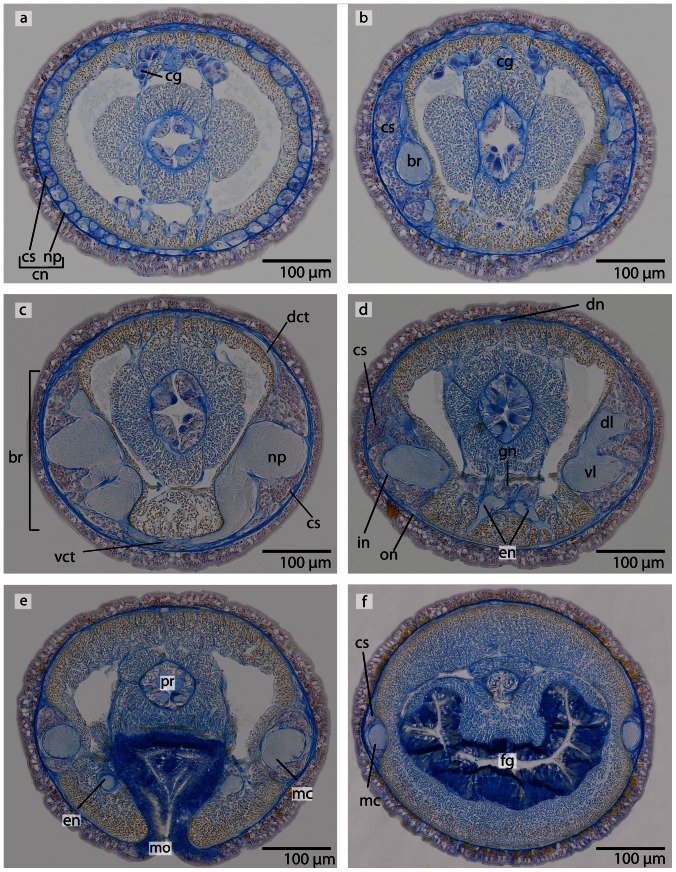
*Callinera grandis*, light micrographs of Azan stained transverse sections of the brain. **a**: The cephalic nerves (*cn*) are composed of neuropil (*np*) and neuronal cell somata (*cs*), a cephalic gland (*cg*) is located dorsally. **b**: The anterior region of the brain (*br*) is covered by a enormous layer of cell somata (*cs*). **c**: The brain is composed of a central neuropil (*np*) and a surrounding layer of cell somata (*cs*). The two halves of the brain are connected by ventral (*vct*) and dorsal (*dct*) commissural tracts. **d**: Posteriorly the brain is divided into a ventral (*vl*) and dorsal (*dl*) section. A dorsal nerve (*dn*) arises from the dorsal commissural tract, and the paired esophageal nerves (*en*) arise from the ventral commissural tract. Shortly anterior to the foregut a concentration of neurons (*gn*) occurs which are associated with the esophageal nerves. The somata are separated from the neuropil by an inner neurilemma (*in*), and the whole brain is enclosed by an outer neurilemma (*on*). **e**: The ventral lobes of the brain are confluent with the lateral medullary cords (*mc*). *mo*: mouth opening, *pr*: proboscis. **f**: The lateral medullary cords (*mc*) run to the posterior of the animal. The cell somata (*cs*) cover the neuropil in a C-shaped manner. *fg* foregut.

**Figure 23:**
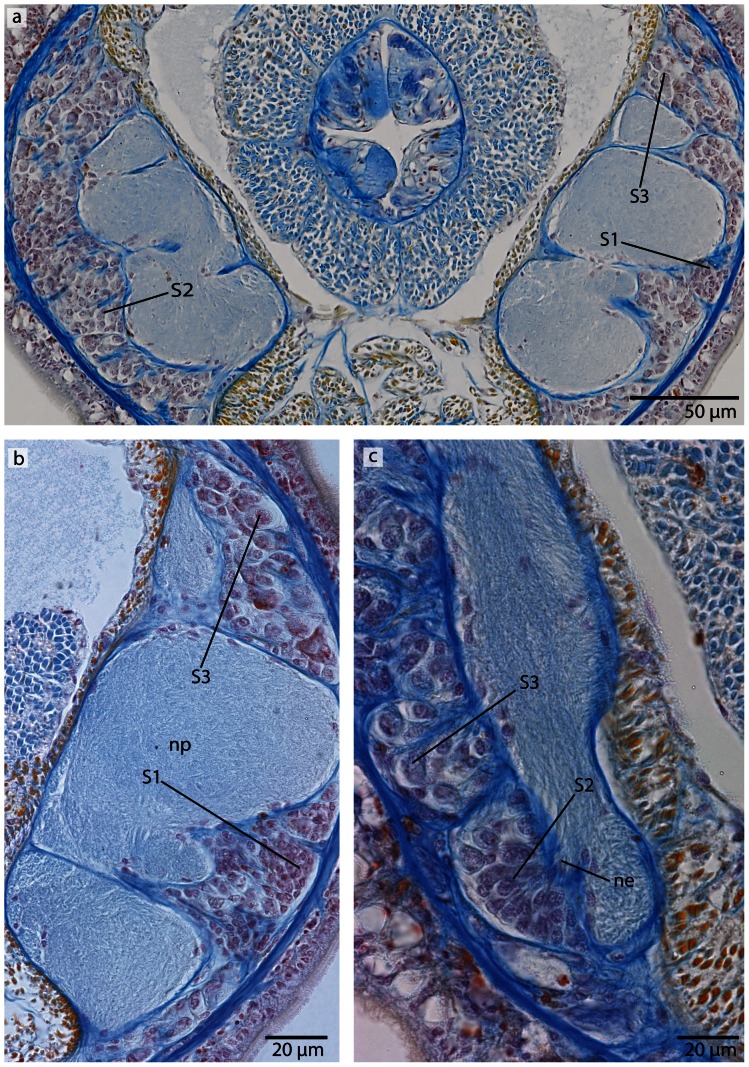
*Callinera grandis*, light micrographs of Azan stained neurons. **a**: Transverse section, overview of the brain; position of the three different types of neurons (*S1*–*S3*); all nuclei stain purple. **b**: Higher magnification of somata of type 1 and 3 neurons (*S1*, *S3*). Cell bodies are circular; those of S1 are arranged in clusters, those of *S3* are most prominent. **c**: Higher magnifcation of somata of type 2 and 3 neurons. *S2* cell bodies are pear shaped and slightly enlarged. The neurites (*ne*) of the brain branch into the cell somata layer. *np* neuropil.

As in the other species studied, the brain is composed of neuropil, which is surrounded by a layer of neuronal cell somata (figs. 21, 22b, c; 23a–c). The entire organ is encased in an outer neurilemma which cannot be differentiated from the epidermal basal lamina, so that the brain is subepidermal (fig. 22c). An inner neurilemma separates the somata of the neurons from the neuropil (fig. 22d). The brain of *Callinera grandis* is located ventro-laterally and close to the mouth opening. Anteriorly the brain is juxtaposed to the head lacuna, merely separated from the blood space by a small muscle layer (fig. 22c). The brain consists of four lobes that are connected by a broad and continuous lateral tract, so that, like in both *Tubulanus* species, dorsal and ventral lobes can hardly be discriminated as distinct morphological structures (fig. 22c). The terms dorsal and ventral lobes, however, will be kept for the remaining description.

In front of the brain, a pair of lateral bundles of neurites gives rise to anteriorly directed cephalic nerves as well as to the proboscidial nerves (fig. 23c). Anterior to the brain, the dorso-medial and the ventral-medial sections of the animal initially contain no obvious neuronal structures. As they travel anteriorly, the dorsal- and ventral-most cephalic nerves branch into additional nerves so that a massive ring of parallel cephalic nerves is established about 40 µm in front the brain (figs. 21, 22a).

The lateral bundle of neurites which gives rise to the cephalic nerve is posteriorly connected to the dorsal lobes and to the ventral lobes. The latter connection, however, is less prominent. Both dorsal lobes are located anterior to the ventral ones and are connected by two small dorsal commissural tracts. The posterior section of the dorsal lobe shows a lobe-like dorsal extension that consists of one posteriorly and one anteriorly directed horn. The latter gives rise to the posterior dorsal commissural tract.

Both ventral lobes are connected by a broad and prominent ventral commissural tract (fig. 22c). On either side, this tract originates where the small bundle of neurites connects the crescent shaped origin of the cephalic nerves to the ventral lobes. About 50 µm caudal to the posterior margin of the ventral commissural tract, three successive, small commissural tracts connect the ventral lobes. The neurites of the ventral commissural tracts are horizontally arranged.

A cluster of neurons occurs at the heights were the two esophageal nerves branch off the minor ventral commissural tract (fig. 22d). These neurons are associated with the esophageal nerves as they follow the course of the latter. The neurons are located dorsally to the nerves and ventrally to the proboscidial muscles and follow the esophageal nerves until they divide into a pair of nerves on each side along the foregut. At this point the neurons have contact to the neurons of the medullary cords.

#### Medullary cords

The ventral lobes taper caudally and give rise to lateral medullary cords. Located directly underneath the epidermal basal lamina, the medullary cords run to the posterior (fig. 22f). Several small rings of neurites connect both medullary cords. The somata of the medullary cord neurons are apposed to the exterior face of the cords to form a lateral, crescent cap (fig. 22f).

#### Neurons

Like in most of the previously described species, the neurons fall into three size classes. Somata and nuclei of class 2 neurons are twice as large as those of class 1 neurons and half as large as those of class 3 neurons (figs. 23a–c). Cell bodies of class 1 neurons are small and circular, with bright red cytoplasms and a purple nuclei (figs. 23a, b) (diameter: 2.82±0.29 µm, n = 17). These cells are primarily found on the dorsal lobe of the brain. Class 2 neurons are pear shaped, have purplish cytoplasms and their nuclei stain slightly darker (figs. 23a, c) (diameter: 4.49±0.62 µm, n = 11). These cells are found on the lateral and ventral parts of the brain. Cell bodies of class 3 neurons stain bright purple, as does the nucleus; their prominent nucleolusis orange (figs. 23a–c) (diameter: 11.26±1.29 µm, n = 12). All size classes differ significantly in diameter (p<0.001).

### Minor nerves and peripheral nervous system

The **cephalic nerves** from a complete ring of longitudinal, partly-interconnected neurites with occasionally interspersed nuclei that are completely isolated from each other by an *ecm* (figs. 21, 22a). Since these nerves are subepidermal, their *ecm* and the epidermal basal lamina form a massive layer of extracellular material frontal to the brain. More than 60 of these nerves can be seen in transversal sections of the head region (fig. 22 a). A **dorsal nerve** arises from the first dorsal commissural tract of the brain. It runs to the posterior, embedded between the basal lamina of the epidermis and a layer of circular muscles (fig. 22d). This nerve is interconnected with the circular bundles of neurites that connect both medullary cords, thus forming a dorsal connection between the rings of neurites. A **ventral nerve** is not present. Each **esophageal nerve** has two roots that arise from the lateral portion of the first two small ventral commissural tracts that posteriorly follow the large and prominent anterior ventral tract (figs. 21, 22e). The roots merge on either side of the animal to form a pair of esophageal nerves, which run dorso-medially to merge anterior to the mouth. Ten to 15 µm posterior to this junction, both nerves separate again and surround the mouth on either side. During their course around the mouth, each esophageal nerve bifurcates. Both branches run in parallel laterally and ventro-laterally on either side of the gut toward the posterior. The origin of the **proboscidial nerves** is unusual, since it arises from the ventral portion of the crescent bundle of neurites, which also gives rise to the cephalic nerves. They initially run dorso-medially, pass the basal lamina of the proboscis epithelium and turn to run posteriorly as basiepithelial nerves.

### Sensory structures

No sensory structures such as eyes or a cerebral sense organ are present in *C. grandis*. An enormous layer of **cephalic glands** is located in the frontal part of the animal, anterior to the brain (figs. 22a, b). These glands are in close contact to the blood lacunae and are connected to the dorsal cephalic nerves, indicating that they might represent neurosecretory structures.

### *Carinina ochracea* (Tubulanidae, “Palaeonemertea”)

**Figure 24:**
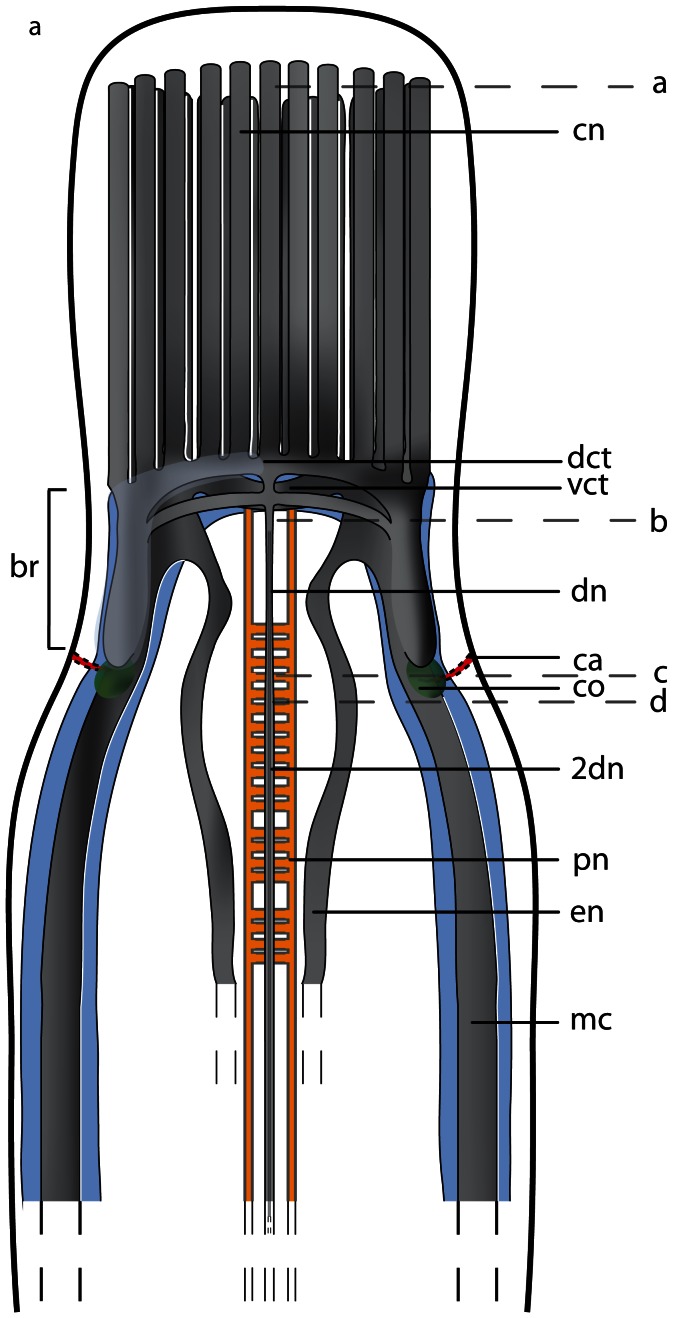
*Carinina ochracea*, schematic drawing of a dorsal view on the central nervous system, based on 3D-reconstruction of 160 aligned 0.5 µm sections. The nervous system is composed of neuropil (*np*, *gray*) which may be surrounded by cell somata (*cs*, *blue*). Cephalic nerves (cn) form basiepidermal basket, the paired proboscidial nerves (*pn*, *yellow*) originate from the ventral commissural tract (*vct*). A dorsal nerve strand (*dn*) originates from the dorsal commissural tract (*dct*). A second dorsal nerve (*2dn*) runs underneath the dorsal nerve. The cerebral organs (*co*, *green*) gain contact to the environment via small canals (*ca*, *red*), which open laterally. The cerebral organ is connected to the dorsal section of the brain. The branching esophageal nerves (*en*) originate from the ventral commissural tract (*vct*). The lateral medullary cords (*mc*) originate in the ventral part of the brain. The letters on the right (**a**–**d**) refer to the histological sections in figure **25**.

The nervous system of *C. ochracea* is basiepidermal; the brain is located anterior to the mouth and is externally visible (figs. 3f, 24), (see: https://www.morphdbase.de?P_Beckers_20130201-M-13.1 for *Carinina ochracea* stack)

### Central nervous system

#### Brain

**Figure 25:**
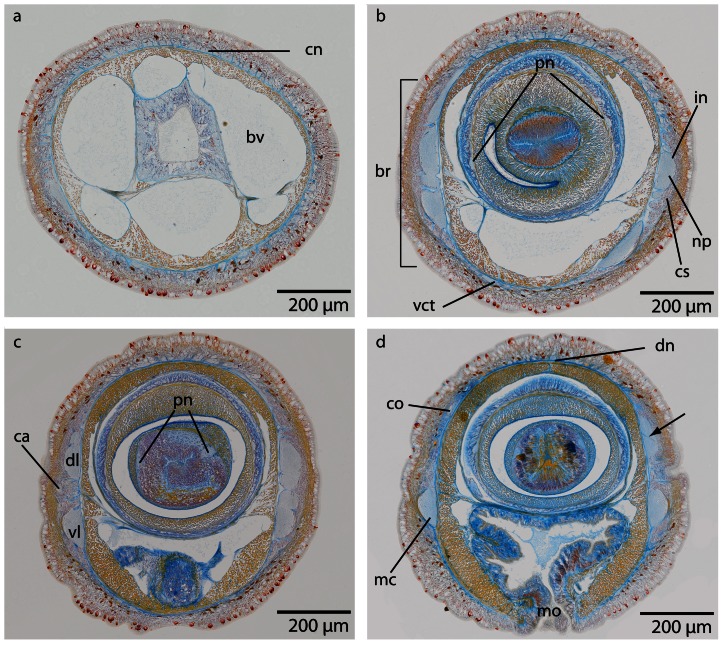
*Carinina ochracea*, light micrographs of Azan stained transverse sections of the brain. **a**: Frontal region showing the circularly arranged cephalic nerves (*cn*). *bv*: blood vessel. **b**: The brain (*br*) is composed of a central neuropil (*np*) which is surrounded by cell somata (c*s*). A ventral commissural tract (*vct*) connects the two halves of the brain, and an outer neurilemma (*on*) encloses the whole brain. (*pn*): proboscidial nerves. **c**: The canals (*ca*) of the cerebral organs open laterally, where the brain divides into a dorsal (*dl*) and ventral (*vl*) sections. Two proboscidial nerves (*pn*) are present. **d**: The canals of the cerebral organs (*co*) end dorsally. A dorsal nerve strand (*dn*) arises from the dorsal commissural tract, the ventral part of the brain is confluent with the lateral medullary cords (*mc*). *Note* the ecm (*arrow*) branching into the neuropil of the brain. *mo* mouth opening.

The brain of *Carinina ochracea* is basiepidermal, resting on the epidermal basal lamina and an outer neurilemma is missing (figs. 24, 25b). The central part of the brain neuropil is partly separated by an inner neurilemma from the neuronal cell somata, which form the outer layer of the brain (fig. 25b). Fibers of extracellular matrix branch into the brain neuropil (fig. 25d). In contrast to the previous species, the brain does not consist of four discernable lobes. Instead, either side of the brain consists of a pair of exteriorly bulged and crescent-shaped lobes (fig. 25b). Both lobes are connected by a small dorsal commissural tract and a broad ventral commissural tract (figs. 24, 25b). The neurites of the commissural tracts are horizontally arranged, with both commissures located on the same level, so that in transverse sections the brain appears to be U- or ring-shaped, depending on whether the dorsal commissure is sectioned or not (figs. 24, 25b, 27a, b). The dorsal commissural tract gives rise to several anteriorly directed and initially interconnected cephalic nerves. The dorso-frontal margin of the brain is distinctly bifurcated with one branch being oriented anterior-ventrally and the other anterior-dorsally. Both branches also give rise to cephalic nerves, but those originating from the anterior-ventral branch are rather short (fig. 25a). A small, globular extension originates from the dorsal-caudal part of each lobe, tapering quickly and ending broadly in a layer of neuronal cell somata. The ventral commissural tract is slightly crescent-shaped, so that its anteriormost point is located further frontally than the dorsal commissural tact. A few short cephalic nerves originate from the anterior margin of the ventral commissural tract.

**Figure 26:**
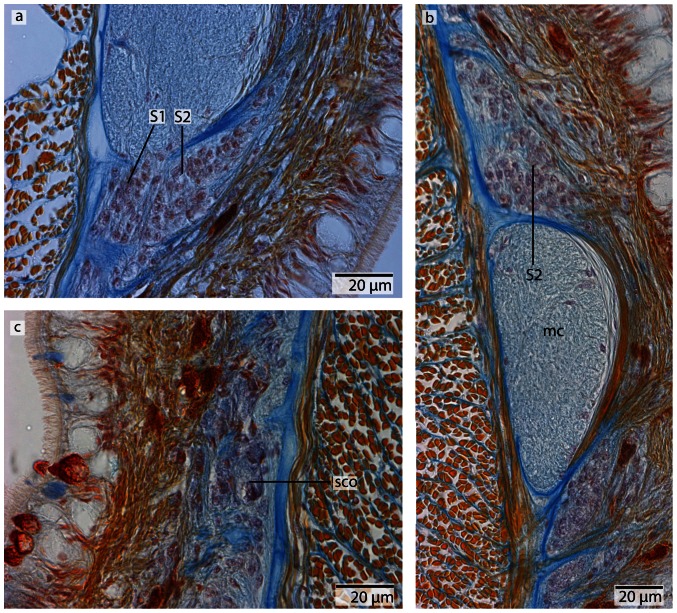
*Carinina ochracea*, light micrographs of Azan stained neurons. **a**: Cell bodies of type 1 neurons (*S1*) are not enlarged while those of type 2 neurons (*S2*) are; nuclei stain purple. **b**: Neuronal cell somata of the medullary cord (*mc*) are of the second type described for the brain. **c**: The canal of the cerebral organ ends in a layer of special neuronal cell somata (*sco*), which are not present in the brain.

#### Medullary cords

The medullary cords arise from the ventral margin of the each brain lobe. They run posteriorly exterior to the epidermal basal lamina and thus are basiepidermal. The neuronal cell somata cover the neuropil of the medullary cords dorsally and ventrally like a cap (fig. 25d).

#### Neurons

**Figure 27:**
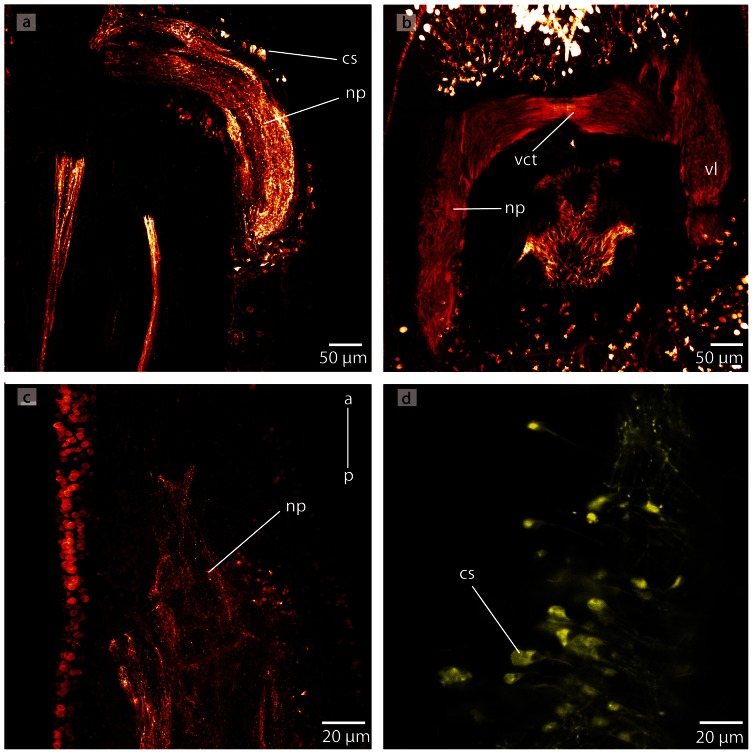
*Carinina ochracea*, confocal laserscanning (cLSM) micrographs of differently immunostained vibratome sections. **a**: Anti-serotonin, the brain is composed of neuropil (*np*) and cell somata (*cs*). Note the few neuronal cell somata showing immunoreactivity against serotonin. **b**: Anti-α-tubulin, the neurites of the neuropil (*np*) of the brain are horizontally arranged in the ventral commissural tract (*vct*). *vl*: ventral lobe. **c**: Anti-serotonin; only few neurites show immunoreactivity against serotonin. **d**: Anti-FMRF. The cell somata (*cs*) may represent glia cells. *a*: anterior *np*: neuropil, *p*: posterior.

Like in *Carinoma mutabilis*, the somata of the neurons fall into two size classes, with somata and nuclei of class 2 neurons being twice as large as those of class 1 neurons (fig. 26). Cell bodies of class 1 neurons are small and circular with nuclei that stain purple (fig. 26a) (diameter: 2.58±0.46 µm, n = 14). The nuclei of class 2 neurons also stain purple (figs. 26a, b) (diameter: 6.11±0.74 µm, n = 10). The different types of neuronal cell somata cannot be discriminated with the applied immunostaining techniques (figs. 27a–d). Class 1 neurons prevail throughout the central nervous system and are characteristic for the medullary cords. A third type of neurons is found around the terminal part of the canal of the cerebral sense organ and will be described below (fig. 26c). The two size classes differ significantly in diameter (p<0.001).

### Minor nerves and peripheral nervous system

The ring of longitudinal **cephalic nerves** initially forms a basiepidermal basket in the animal's head (figs. 24, 25a). The dorsal cephalic nerves originate from the dorsal commissural tract, the lateral ones from both anterior branches of the dorsal lobe and the ventral ones from the ventral commissural tract. All cephalic nerves are initially interconnected. As they travel, they become separated from each other by an *ecm* (figs. 24; 25a). The lateral cephalic nerves are much shorter than the dorsal ones, so that merely dorsal and ventral cephalic nerves are found in the anterior part of the head. A **dorsal nerve** arises from the dorsal commissural tract and runs posteriorly, directly opposed to the epidermal side of the basal lamina (basiepidermal) (fig. 25d). A second dorsal nerve runs posteriorly underneath the *ecm of* the rhynchocoel. Both dorsal nerves are repeatedly interconnected by small neurites. A **ventral nerve** is not present. Posterior to the ventral commissural tract, two **esophageal nerves** branches off of each ventral lobe. Both esophageal nerves turn inward, surround the mouth and run posteriorly on either side of the foregut. Two proboscis nerves arise from the posterior margin of ventral commissural tract (figs. 24, 25b). These nerves run posteriorly on either side of the proboscis. A proboscidial plexus regularly interconnects both nerves.

**Figure 28:**
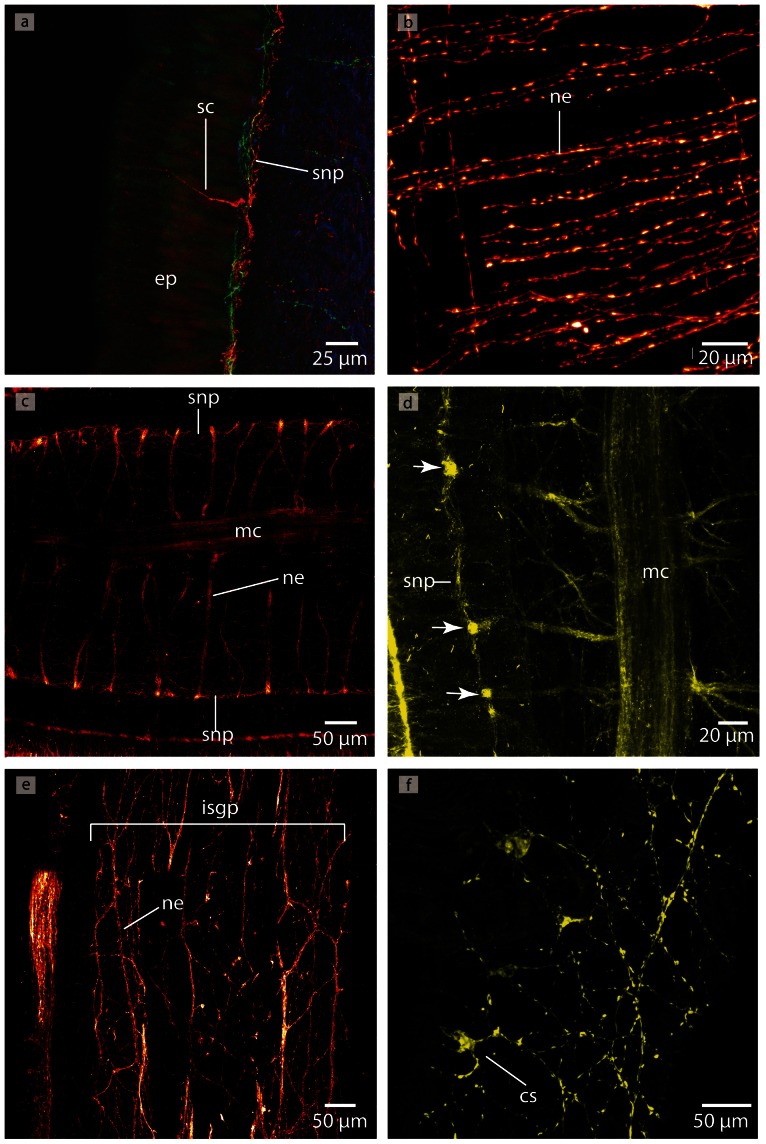
*Carinina ochracea*, confocal laserscanning (cLSM) micrographs of differently immunostained vibratome sections. **a**: Anti-FMRF, horizontal section. A sensory cell (*sc*) originates in the subepidermal plexus (*snp*) and proceeds into the epidermis (*ep*). **b**: Anti-FMRF, horizontal section. The neurites (*ne*) of the intraepidermal nerve plexus are arranged in a ladder-like manner. **c**: Anti-α-tubulin, sagital section. The lateral medullary cords (*mc*) are interconnected to the subepidermal plexus (*snp*) by branches of neurites (*ne*). **d**: Higher magnification of **c**. Note the concentration of neurites in the subepidermal nerve plexus (*arrows*) **e**: Anti-FMRF, horizontal section. The neurites (*ne*) of the intrastomatogastric nerve plexus are irregular arranged. **f**: Anti FMRF, horizontal section. Multipolar neurons (*cs*) are distributed in the intraepidermal nerve plexus.

Several different **nerve plexus** are present in *C. ochracea* (figs. 28a–e). The neurites of the intraepidermal plexus show immunoreactivity with an antiserum directed against FMRFamid and form a ladder-like meshwork (fig. 28b). A subepidermal nerve plexus becomes visible when stained with an antiserum directed against FMRFamid and against α-tubulin (figs. 28a, c, d). Distinct spherical neurite concentrations within this plexus give rise to neurites that connect it to the lateral medullary cords (figs. 28c, d) Whether these are circular or not could not be determined due to the size of the animals and the methods used. The neurites of the intrastomatogastric nerve plexus show a diffuse net-like pattern (fig. 28e). A commissural or a rhynchocoelan plexus was not discernable.

### Sensory structures

#### Cerebral organ

**Figure 29:**
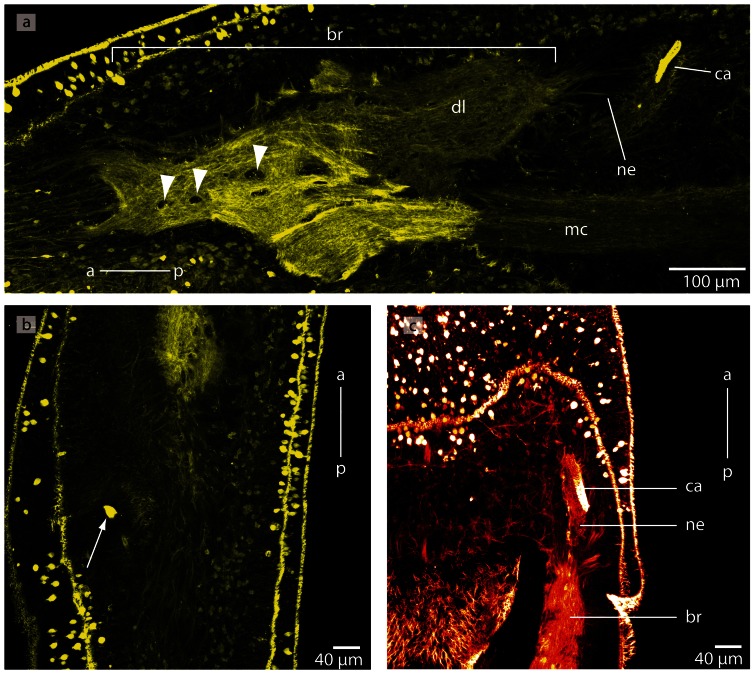
*Carinina ochracea*, confocal laserscanning (cLSM) micrographs of anti α-tubulin immunostained vibratome sections. **a**: Sagital section. The neurites (*ne*) of the cells lining the canal of the cerebral organ are connected to the neuropil of the dorsal lobe (*dl*) of the brain (*br*). Note the unstained structures in the neuropil of the brain (*arrowheads*). **b**: sagital section. The canal terminates in a pit with a great number of cilia (*arrow*). **c**: horizontal section. The neurites (*ne*) of the sensory cells built a dense meshwork which is connected to the brain (*br*). *ca* canal of the cerebral organ, *mc* medullary cord.

The cerebral sense organs are paired, tube-like structures or ducts that extend inward and terminate dorsally above the dorsal globular extension of the brain. They originate on either side of the animal from a shallow epidermal pit that is located on the level of the terminal section of the dorsal lobe (figs. 24; 29a–c). The cerebral organ initially runs medially, enters the dorsal lobe's neuronal somata, turns dorsally and runs posteriorly for a few micrometers finally passing the somata layer to blindly end dorso-laterally (figs. 25d, 29a). The entire cerebral organ is lined by ciliated and sensory cells; the density of the latter, however, increasing towards the inner part of the organ. Immunostaining with anti-α-tubulin shows a very brightly stained structure, which presumably represents densely arranged cilia (figs. 29b, c). Azan staining reveals cell somata that differ from those of the brain and to those of the medullary cords (fig. 26c), since they are elongated, twice as large as class 2 neurons and their nuclei stain purple. Anti- α-tubulin immunostaining also shows that the sensory cells are connected to the posterior part of the dorsal lobe of the brain by small branches of neurites (figs. 29a–c). There is no contact between the cerebral organ and the blood vessels. Pigmented eyes and frontal organs are absent in this species.

#### Sensory cells

Many sensory cells are distributed all over the animal. Most of these intraepidermal cells originate from the subepidermal nerve plexus (fig. 28a). The cells presumably have a tactile function.

## Discussion

Most textbooks simplify the nemertean central nervous system as being quite uniform, consisting of a four lobed brain and two lateral medullary cords [5], [27], [28], [29], while others describe the nemertean nervous system as being primarily ring-shaped [30]. The present study, however, shows a remarkable diversity in the organization of the central nervous system among palaeonemertean species and corroborates a previous comparative analysis [31]. While the *cns* is strictly bilaterally symmetrical, four lobes can clearly be identified in only in a few species. In addition, the peripheral nervous system also differs amongst different groups and often even between closely related species. Palaeonemerteans most likely represent a grade of basally branching nemertean groups [4], [5], [14], [15], [16], but this is not unanimous [17]. Comparing their nervous systems, thus, could reveal some important insights into the primary organization of the nemertean nervous system. The present study covers representatives of the three larger palaeonemertean groups *Cephalothricidae*, *Carinomidae* and *Tubulanidae*. Since the monophyly of the latter is questionable [32], we analyzed representatives of each genus except for *Carinesta*. In the subsections below we will comparatively evaluate the central nervous system in nemerteans in terms of topology, structure, types of neurons and extracellular components as well as their peripheral nervous system and their sensory organs.

### Brain and central nervous system

#### Topological position

The topological position of the central nervous system (*cns*) differs between the palaeonemertean species. Like in other cephalothricids [4], [31], [33], [34], [35], [36] the *cns* is located inside the musculature of both cephalothricid species studied. There is only very limited information on the nervous system of carinomids, however, Friedrich [37], mentioned that the anatomy of carinomids is rather uniform throughout the group, so we assume the intramuscular position of the *cns* in *Carinoma mutabilis* is characteristic for Carinomidae. The course of the medullary cords, however, differs between carinomids and cephalothricids. While they are completely intramuscular and run within the longitudinal muscle layer in cephalothricid species, this is only the case in the posterior region of carinomid species; anteriorly they are situated underneath the outer circular muscle layer. This observation contradicts [38] who claims that the medullary cords are initially located outside the circular muscles in *Carinoma* species. In *Tubulanus*, *Callinera*, and *Carinesta* species, the brain and medullary cords are subepidermal, located between the subepidermal *ecm* and the underlying outer circular muscles [31], [34], [35], [36], [38], [39]. In *Carinina ochracea*, like in other *Carinina* species they are found basiepidermally [31], [36], [38].

Bürger [4] and Iwata [18] assumed the position of the *cns* as having important implications for considerations of nemertean phylogeny. Bürger [4] supposed that a basiepidermal nervous system, like that of *C. ochracea* was characteristic for the nemertean ancestor and assumed that the *cns* migrated inward during nemertean evolution, so that a subepidermal nervous system represents a derived condition in Nemertea. Iwata [13], on the other hand, supposed the Cephalothricidae to be the basal most branching taxa, due to the location of the *cns* inside the musculature and the lack of a cerebral organ. This interpretation suggests that any other topological position of the *cns* must therefore be derived.

Presently, we are unable to provide support for either traditional interpretation of *cns* evolution since the position of nemerteans within the Spiralia is unresolved and any position of the *cns* found within the nemerteans can also be found in at least one of the possible outgroups. In platyhelminthes [40], sipunculids [41] and kamptozoans [42], the nervous system is located subepidermally. In basal molluscs [43] the nervous system is located in the longitudinal musculature and in brachiopods [44] and phoronids [45] it is located basiepidermally. In annelids the nervous system may be located basiepidermally (*Owenia fusiformis*, [46]), or in the musculature (*Nereis* species, [47]). Despite an ongoing debate about the phylogeny of annelids [48], [49], the heterogeneous position of the nervous system within the Spiralia needs a thorough cladistic analysis. The presently known topological differences, however, may indicate that this character does not contain a signal for unraveling the higher-level phylogeny.

#### Structure

A brain lobe has been defined above as voluminous compartment of the brain that is connected to other such compartments by tracts. The heteronemertean *L. viridis* has paired dorsal and ventral lobes, each of which is more or less completely surrounded by a matrix and thus a clearly identifiable morphological unit [24]. Identification of these lobes is difficult, if such an extracellular surrounding is missing. Despite the lack of an *ecm* that demarcates the lobes, however, their larger volume compared to the commissural and lateral tracts allows identification of all four brain lobes in non-tubulanid palaeonemerteans. In cephalothricid species, several lateral tracts connect dorsal and ventral lobes on each side of the brain. In *Callinera grandis*, and in both of the *Tubulanus* species examined, dorsal and ventral lobes from each body side are connected by an anterior-posterior broad, but medio-laterally narrow lateral tract, so that slight differences in the volume allow the identification of all four brain lobes. This is especially evident, when taking a look at 3D-reconstructions of the brain. If these are not available, some ambiguity will remain when describing the structure of the brain and lead to differing descriptions of the brain in different species of the same genus (see table 1).

**Table 1:** Elements of the nervous system of basally branching nemerteans.

taxon	lateral lobe	dorsal lobe	ventral lobe	lateral tract	dorsal commissure	ventral commissure	cephalic cords	medullary cords	cephalic nerves	dorsal nerve	2^nd^ dorsal nerve	ventral nerve	esophag. nerve	probscid. nerve	cerebral organ	Author
*Cephalothrix linearis*	-	1 pair	1 pair	5 pairs	1	1	4	1 pair	-	1	-	1	-	2	-	this study
*Procephalothrix filiformis*	-	1 pair	1 pair	5 pairs	1	1	4	1 pair	-	1	-	1	-	2	-	this study
*Carinina ochracea*	1 pair	-	-	1 pair	2	1	-	1 pair	>5-	1	1	-	2	2	1 pair	this study
*Tubulanus superbus*	-	1 pair	1 pair	1 pair	>5	1	-	1 pair	>5-	1	-	-	2	2	1 pair	this study
*Tubulanus polymorphus*	-	1 pair	1 pair	1 pair	>5	1	-	1 pair	>5-	1	-	-	2	2	1 pair	this study
*Callinera grandis*	-	1 pair	1 pair	1 pair	2	1	-	1 pair	>5-	1	-	-	2	2	-	this study
*Carinoma mutabilis*	-	1 pair	1 pair	1 pair	3	1	-	1 pair	>35	1	-	-	2	2	-	this study
*Carinina grata*	-	1 pair	1 pair	pair	1	1	-	1 pair	?	1	-	-	2	2	1 pair	[4]
*Carinina johnstoni*	1 pair	-	-	-	1	1	-	1 pair	?	1	-	-	2	?	1 pair	[36]
*Carinesta dellechiajei*	1 pair	-	-	-	1	1	-	1 pair	?	1	-	-	1	?	-	[36]
*Callinera blanchardi*	1 Pair	-	-	-	1	1	-	1 pair	several	1	-	-	2	2	-	[36]
*Callinera quatrefagesi*	1 pair	-	-	-	2	1	-	1 pair	several	1	-	-	2	?	-	[36]
*Cephalotrichella gaimardi*	-	1 pair	1 pair	,	1	2	-	1 pair	4	1	-	-	2	2	-	[36]
*Carinesta orientalis*	1 pair	-	-	-	2	2	-	1 pair	>5-	1	-	-	2	?	-	[39]
*Tubulanus lucidus*	-	1 pair	1 pair	1 pair	?	1	-	1 pair	?	1	-	-	2	?	1 pair	[34]
*Carinesta uchidai*	1 pair	-	-	+	?	1	-	1 pair	?	1	-	-	2	?	-	[34]
*Procephalothrix fasciculus*	-	1 pair	1 pair	+	1	1	4	1 pair	-	1	-	1	-	?	-	[34]
*Procephalothrix arenarius*	-	1 pair	1 pair	+	1	1	4	1 pair	-	1	1	1	-	?	-	[35]
*Carinesta tubulanoides*	1 pair	-	-	-	1	1	-	1 pair	+	1	1	-	?	2		[35]
*Tubulanus norvegicus*	1 pair	-	-	-	1	?	-	1 pair	?	1	1	-	2	?	-	[73]

In *Carinina ochracea* however, no criteria exist to identify dorsal and ventral lobes. Instead the entire brain consists of two laterally bulged lobes that are connected by a dorsal and at least one ventral commissural tract. The same has been stated for *Carinesta* species [39], [36]. Given that tubulanids are a basal grade within the nemerteans, as indicated by some molecular studies [16], [17], the bi-lobed, ring-shaped brain found in *Carinina* and *Carinesta* species [39], [50], [31], [36] could represent the primary condition of the nemertean brain. Moreover, its basiepidermal position in *Carinina* would then be in accordance with Bürger's [4] assumption of such a position representing a primary condition in nemerteans. This assumption implies that the brain becomes increasingly regionalized with its dorsal part becoming prominent and separated during the nemertean evolution – an evolutionary scenario that has already been argued for eloquently by [31]. This scenario requires interpretation of the brain structure in *Callinera* and *Tubulanus* as intermediates. The bi-lobed, ring-shaped brain without clearly identifiable dorsal and ventral lobes in *Carinina* species could, however, also be explained by condensation of the brain and loss of brain compartments, making the brain structures in *Callinera* and *Tubulanus* again intermediates. Although this alternative explanation presently seems unlikely to us (see below), a final decision will require a cladistic analysis of the nemerteans that includes additional anatomical characters. Complex internal compartmentalization, like the one found in a number of annelid representatives that contain mushroom bodies, has not been observed in nemerteans. [51], [52]

Medullary cords have been described in Platyhelminthes [40], basal molluscs [53], [54], [55], [56], [57] and polychaetes [47], [58]. The arrangement of the neuronal cell somata surrounding the neuropil of the medullary cords is not described in these studies, although they are present according to classical contributions [40], [43], [47]. As longitudinally arranged nerve cords, these parts of the nervous system can certainly be homologized, but it remains to be resolved which cords of the tetraneural mollusk and the orthogonal plathelminth nervous system are homologous to the nemertean medullary cords. This, as well as an evaluation of the polychaete medullary cords needs thorough cladistic analyses based on morphological matrices that contain detailed information on the nervous system. A serial arrangement of circular nerves connecting the medullary cords is found in *Procephalothrix filiformis*. In *Carinina ochracea*, the neuropil of the medullary cords is connected to the neurites of the subepidermal nerve plexus with regular arranged branches of neurites showing immunoreactivity against α-tubulin. In addition, at the spots where the neurites of the subepidermal nerve plexus branch off, a spherical concentration of neurites occurs. Since a serial arrangement of particular nerves is also present in platyhelminthes [40], annelids [47] and basal molluscs [43], we suggest this as an element of the last common ancestor of the Spiralia or a subgroup within this clade.

#### Neurons

The neuronal cell somata surrounding the brain neuropil appear to be different in all investigated palaeonemerteans. Despite their different staining, the neurons of palaeonemertean species fall in three size classes, with the second and the third being almost twice as large as their previous ones. The third, largest class, which is missing in *Carinina ochracea*, always possesses a prominent nucleolus and a large nucleus. Its morphology implies high metabolic rates. The first class of cells (type 1 neurons) predominates in all nervous systems. Neurochord cells (type 4 according to Bürger [4]) are missing in the palaeonemertean groups except for *Cephalothricella gaimardi* [36]. Although Bianchi [59] discriminated six types of neuronal cell somata in the heteronemertean *Cerebratulus marginatus*, the three types of neuronal cell somata in *Lineus viridis* (Heteronemertea) described by [25] most parsimoniously represent the primary condition in nemerteans. Since the neurons in basal mollusks have also been grouped in three classes by size differences [60], this could hint at an ancient pattern in nerve cell organization in Spiralia. Presently, the data are too scattered to draw any conclusion, but it may be fruitful to include neuron size into a cladistic analysis.

#### Neurilemma

The neuronal cell somata of the brain may be separated from the neuropil by an extracellular matrix, also termed inner neurilemma [4]. Within the Cephalothricidae, *Carinina ochracea* has an inner neurilemma, while it is lacking in *Callinera grandis* and *Tubulanus*. Little is known about the function of the extracellular matrix in the invertebrate nervous system. This *ecm* may be involved in neurite formation, maintaining structure and function as well as in repair processes [61], [62]. An extracellular matrix surrounding or encapsulating the brain has been described for basal molluscs [53], for certain platyhelminthes [40] and polychaetes [47]. Although this seems to indicate that an *ecm* surrounding the brain is characteristic either for a common stem species of the Spiralia or for a group within the Spiralia including annelids, mollusks and nemerteans, the outer neurilemma cannot be differentiated from the epidermal basal lamina in basiepidermal and subepidermal nervous systems. The outer neurilemma, thus, might be nothing more than a reinforced subepidermal matrix and therefore overestimated in evolutionary considerations. Nevertheless, an inner neurilemma, which is present in some palaeonemertean species, is unique and seems to be of phylogenetic importance.

### Minor nerves and peripheral nervous system

Cephalic nerves differ in their arrangement and position between taxa. In Cephalothricidae there are four cephalic cords; cephalic nerves were not found. In the remainder of the investigated palaeonemerteans (*Tubulanus*, *Callinera grandis*, *Carinoma mutabilis*, *Carinina ochracea*), they are circularly arranged around the head lacuna. Since the heteronemertean *Lineus viridis* also possess an anterior ring of several cephalic nerves, such an organization must represent a primary condition in nemerteans, making the nerve cords in cephalothricid species derived, probably correlated with the expansion of the preoral body region that characteristic for this group.

A dorsal nerve is present in all palaeonemertean species. The dorsal nerve of Cephalothricidae and *Carinoma mutabilis* originates in the dorsal commissural tract inside the musculature. The nerve then runs dorsally and finally lies just underneath the basal lamina of the epidermis. A ventral nerve is only present in Cephalothricidae. It has two roots that branch off the ventral lobe at the origin of the ventral commissure, like the esophageal nerves of the remaining palaeonemertean species. The ventral nerves encircle the mouth like the esophageal nerves, however they differ by running below the gut as single nerve. Since a ventral nerve is lacking in the remaining palaeonemerteans and esophageal nerves are lacking in Cephalothricidae, the origin, position and initial course clearly indicate a homology of the ventral nerve and the esophageal nerves. This hypothesis is supported by Gibson's [35] remark that the ventral nerve (foregut nerve) supplies the foregut. All investigated palaeonemerteans possess paired proboscidial nerves, which also originate in the ventral commissural tract of the brain.

Palaeonemerteans possess nerve plexus in different parts of their body. These plexus may be well developed and can clearly be shown by immunohistochemistry. The arrangement of the neurites found there differs between nerve plexus but not between species.

### Sensory structures

Nemerteans perceive chemical substances with different chemo-sensitive sensory structures, which are situated on the head of the animals. These are called the frontal and cerebral organs. As shown in studies of a hoplonemertean [11] and cephalothricid palaeonemertean [12], these sensory structures are designed for a hunting life style. Frontal organs are not present in the species investigated here, although they have been described for *Procephalothrix simulus* (Palaeonemertea) [63].

The cerebral organs of *Tubulanus* species consist of simple epidermal tubes that open laterally to the environment and terminate exterior to the basal lamina in the epidermis. The inner part of the tube's epithelium is lined with sensory cells, which are connected to the brain. In *Carinina ochracea*, the canal of the cerebral organ is elongated and runs dorsally but it also terminates exterior to the basal lamina of the epidermis. In contrast to the conditions found in *Tubulanus*, the duct of the cerebral organ in *C. ochracea* passes through the neuronal cell somata of the brain since the brain in *C. ochracea* is basiepidermal. Because of the similarities in the morphology and staining affinities, we assume a common origin of the cerebral organ and not convergent evolution.

In *Carinina ochracea*, several sensory cells originate in the subepidermal plexus and proceed into the outer margins of the epidermis. These cells may have a tactile function. In *Procephalothrix filiformis*, a cluster of cells showing immunoreactivity against an antiserum directed against FMRFamid is present in the tip of the animal's head. These cells might represent sensory cells.

Ciliated sensory structures that may have a chemo-sensitive function are found in Platyhelminthes, Mollusca and in Annelida. Platyhelminthes possess ciliated sense organs on the tip of the head, which are supposed to have a chemoreceptive function [30]. In most annelids species, nuchal organs are present. These are small chambers situated on the prostomium of the animals. The chamber is lined with sensory cells that are connected to the brain by neurites [64], [65], [66]. Aplacophoran molluscs possess a sensory organ on the tip of their head that is also supposed to have a chemoreceptive function [67]. The function, morphology and position of these sensory structures are comparable to the frontal organs of nemerteans. In older studies, a connection between the frontal organs and brain has been described [5], but the present study failed to analyze this connection in detail, because neither Azan nor in the immunostaining revealed sufficient information. The homology of the cerebral organs within the nemerteans is still a matter of debate [16], due to strong positional differences among the taxa. If they are homologous however, the plesiomorphic position would be posterior to the brain, since an anterior position is only described for hoplonemertean species and according to recent phylogenetic analysis [16], [17], Hoplonemertea are derived within the Nemertea. Specific correspondences between the cerebral organs and cephalic sensory organs in other spiralian species are thus far unknown, since innervations pattern and ultrastructural details are largely unknown for the cerebral organs.

## Conclusions

Our comparative survey of the palaeonemertean nervous system reveals that each brain consists of two halves in which the neuronal perikarya (cell bodies, somata; Ganglienzellkörper [36], Ganglienzellen sensu [4]) are opposed and form a layer of neuronal somata. Lateral fiber masses (neuropil), can be discriminated into dorsal and ventral compartments, each of which is called a lobe. Our study also gives rise to the idea of an increasing compartmentalization of the nemertean brain during the course of evolution leading to prominent brain lobes in Hoplo- and especially Heteronemertea. This had two evolutionary consequences: (1) the medial portion of fiber masses became smaller and partitioned, so that it is identified as lateral tracts rather than being part of a large lateral fiber mass, and (2) the lobes dominate the histological section in such a way that each lobe has been termed ganglion by some researchers [4], [34]. Especially in heteronemerteans, this impression is strengthened by the fact that the lobes are separated from each other by a neurilemma. This idea, however, depends on the phylogenetic position and the mono- or paraphyly of the palaeonemerteans.

If the palaeonemerteans were monophyletic as implied by recent molecular studies [17], the idea of an increasing compartmentalization of the brain and the translocation of the nervous system into deeper tissue layers during nemertean evolution would either have to be modified or cannot be held: either (1) the intraepidermal nervous system with a rather simple brain represents the primary condition in nemerteans so that compartmentalization and translocation of the nervous happed within the palaeonemerteans and in the stem lineage of Neonemertea or (2) the nervous system was primarily subepidermal with a compartmentalized brain and underwent a translocation and simplification within the palaeonemerteans. In the latter case our idea of an increasing compartmentalization cannot be held upright. The former case requires assuming that brain compartments and translocation of the nervous system into deeper tissue layers evolved repeatedly within nemerteans and, thus, is presently not parsimonious.

Given that palaeonemerteans are a basally branching grade, as implied by older molecular studies [15], [16] and presently missing morphological support for a taxon Palaeonemertea, the nemertean brain primarily consisted of two bilaterally symmetrical lobes, interconnected by a ventral and one or several small dorsal commissures. A pair of medullary cords originates from the ventral portion of the brain. According to this assumption the primary organization of the nemertean nervous system is retained in *Carinina ochracea*. Provided that the brain expanded dorsally and became dorsally commissurized along with the evolution of a proboscis, nemerteans and their spiralian sister taxa should have had at least two longitudinal medullary cords connected by frontal commissures that were laterally reinforced and, thus, resemble the nervous system of basally branching mollusks and annelids. Actually, basiepidermal nervous systems are known from polychaete annelids, phoronids and brachiopods, the molluscan nervous system is always subepidermal (intramuscular). The phylogenetic position of the nemerteans is still unresolved among spiralians and lophotrochozoans [68]. In order to complete the information and to solve this potential conflict, we are presently analyzing the nervous systems of the two remaining subgroups, the Pilidiophora (Heteronemertea+Hubrechtidae) and the Hoplonemerteans and potential outgroup taxa, using the same methods and providing a coding scheme. A final cladistic analysis will help to elucidate the evolution of the nemertean nervous system and test the different hypotheses on its primary design.

## Materials and Methods

### Animals

*Carinoma mutabilis* Griffin, 1898 was found in March 2007 at Friday Harbor, Cattle Point, San Juan Island, WA, USA. *Procephalothrix filiformis* Johnston, 1828 was found on the isle of Sylt in February 2009. *Callinera grandis* Bergendal 1903 and *Carinina ochracea* Sundberg, Chernyshev, Kajihara, Kanneby, Strand 2009 were found in Pouldohan/Brittany France in September 2009. *Cephalothrix linearis* Rathke, 1799; *Tubulanus polymorphus* Renier 1804; *Tubulanus superbus* Kölliker 1845 were found in Roscoff/Brittany France in March 2010.

We neither used endangered species nor were the investigated animals collected in a protected area. All animals were collected with the permission of the local marine biological stations.

### Histology

One or two specimen of each species were anesthetized in a refrigerator using a solution of 7.5% MgCl_2_ in seawater, and fixed overnight in Bouin's fixative modified after Dubosque- Basil. The animals were completely dehydrated in an ethanol series, followed by incubation in methylbenzoat and butanol. Afterwards the animals were preincubated in Histoplast (Thermo Scientific, Dreieich, Germany) at 60°C for three days with several medium changes and finally embedded in Paraplast (McCormick Scientific, Richmond, USA). Different incubation times were used depending on the size of the animal.

Sections 5 µm thick were made using a Reichert-Jung Autocut 2050microtome (Leica, Wetzlar) and transferred to glass slides coated with albumen-glycerin.

Sections were stained with Carmalaun, subsequently differentiated with sodium phosphotungstate (5%), washed in distilled water and stained with aniline blue orange G. Afterwards the sections were embedded with Malinol (Waldeck, Münster, Germany).

Azan stains the neuropil of the nervous system gray. The nuclei of the neuronal cell somata stain red, sometimes with a tinge of orange or purple. The extracellular matrix (*ecm*, called neurilemma in the central nervous system) stains blue, while musculature stains orange.

### Immunohistochemistry

Specimen of one species of each genus (table 2) except for *C. grandis* and *C. mutablis* due to the lack of material were anesthetized in a refrigerator using a solution of 7.5% MgCl_2_ in seawater. The worms were fixed overnight in a 4% paraformaldehyde (Electron Microscope Sciences, Hatfield, PA) in seawater (0.1 M) solution. After fixation, the head or middle regions of the animals were washed several times in cold phosphate buffered saline (PBS 0.01 M). They were then embedded in a gelatine/albumin medium and the blocks were hardened overnight in a 14% formalin solution in the refridgerator. Each sample was cut into sections 60 µm thick with a vibratome (Micron HM 650 V, Thermo Scientific, Dreieich, Germany). The sections were then washed 5 times for 20 minutes in PBS with 0.1% Triton X-100 (Sigma Aldrich, St. Louis, MO, USA) and pre-incubated overnight in a blocking solution of PBS (0,01 M) containing 0.5% TX and 5% normal swine serum (Jackson ImmunoResearch, West Grove, PA). Primary antibodies were added into the blocking solution and incubated overnight at room temperature. The primary antibodies anti- FMRFamide (ImmunoStar, Hudson, WI) and anti-serotonin (Sigma-Aldrich, Saint Louis, MO, USA) were both used at a dilution of 1∶10000. Antibodies against acetylated α-tubulin (Sigma Aldrich, St. Louis, MO, USA) were used at a dilution of 1∶500. After incubation with primary antibodies, the sections were again washed 5 times for 20 minutes in PBS with 0.1% TX and incubated overnight with the secondary antibody conjugated to fluorophore (Cy3- conjugated goat anti-rabbit; Jackson ImmunoResearch, West Grove, PA) at a dilution of 1∶2000 in PBS containing 0.5% TX and 1% normal swine serum. Secondary antibodies against acetylated α-tubulin (Alexa 633, Life Technologies, Darmstadt) were added at a dilution of 1∶250. Subsequently the sections were rinsed again in several changes of PBS containing 0.1% TX and then mounted on chrome alum/gelatine-coated glass slides under glass cover slips using Elvanol (mounting medium after [69]).

**Table 2:** Staining protocol of the palaeonemertean species investigated.

Species	Histology	Immunostaining		
	Azan	α-Tubulin	FMRF	Serotonin
*Procephalothrix filiformis*	x		x	X
*Cephalothrix linearis*	x			
*Carinoma mutabilis*	x			
*Tubulanus superbus*	x			
*Tubulanus polymorphus*	x	x	x	X
*Callinera grandis*	x			
*Carinina ochracea*	x	x	x	x

For whole animal immunostaining, animals were relaxed in a 7.5% MgCL_2_ solution and fixed overnight in 4% paraformaldehyde (Electron Microscope Sciences, Hatfield, PA). Animals were postfixed for 20 minutes in methanol and then washed in several steps of PBS (0,01 M). Afterwards they were washed with PBS containing Triton X at a dilution of 0.1% and preincubated in a blocking solution with NSS (Jackson ImmunoResearch, West Grove, PA). The primary antibodies for anti- FMRFamid and serotonin were added at a dilution of 1∶5000. After the incubation, the primary antibodies were washed out with PBS containing Triton X at a dilution of 0.1%. The secondary antibodies (Cy3 for anti- FMRFamid and anti Serotonin, and Alexa 633 for α-tubulin) were added at a dilution of 1∶2000 and incubated for 24 hours. Afterwards the animals were washed in PBS containing 0.1% Triton X and pure PBS. Then the animals were mounted in Murray clear (2 parts benzyl benzoate, 1 part benzyl alcohol) after having been treated with methanol and isopropanol.

### Analysis and 3D reconstruction

Azan stained slices were analyzed with an Olympus microscope (BX-51) and photographed with an Olympus camera (Olympus cc12), which was equipped with the dot slide system (2.2 Olympus, Hamburg). Afterwards the slices were aligned with imod [70] and imod align (http://www.q-terra.de/biowelt/3drekon/guides/imod_first_aid.pdf). 3D reconstructions were performed with Fiji (1.45b) [71]/Trakem [72] and Amira (4.0). The schematic drawings were performed according to the 3D reconstructions with Illustrator and Photoshop (CS4).

Immunochemical treated samples were analyzed with a Leica confocal laser scanning microscope (TCSSPE excitation wavelength 488 nm, detection range 500–630 nm was used to detect Alexa 488 fluorescence. Excitation wavelength 543 nm, detection range 555–700 nm was used to detect Cy3 fluorescence. Excitation wavelength 633 nm, detection range 650–800 nm was used to detect Alexa 633 fluorescence) using the LAS AF 1.6.1 software. Stacks were loaded into Fiji (ImageJ 1.45k plugin) [71] and further processed, using the “maximum projection” tool of the ImageJ software (Macophonics 1.44p). The images were finally processed with Fiji [71] and Adobe Photoshop (CS4). Figures were arranged using Adobe Illustrator (CS4).

### Statistical analysis

Neuron diameters were measured with Cell*D (3.2 Olympus). For analyses, the SPSS 15.0 statistical package was used. Data of each species and each class of neurons were tested for normal distribution with Shapiro-Wilk test. Since the data were normally distributed, parametric statistics (ANOVA) were used to test whether the size classes of each species differ significantly.
